# Metallic W/WO_2_ solid-acid catalyst boosts hydrogen evolution reaction in alkaline electrolyte

**DOI:** 10.1038/s41467-023-41097-w

**Published:** 2023-09-02

**Authors:** Zhigang Chen, Wenbin Gong, Juan Wang, Shuang Hou, Guang Yang, Chengfeng Zhu, Xiyue Fan, Yifan Li, Rui Gao, Yi Cui

**Affiliations:** 1 i-lab, Vacuum Interconnected Nanotech Workstation (Nano-X), Suzhou Institute of Nano-Tech and Nano-Bionics, Chinese Academy of Sciences, Suzhou, China; 2 School of Materials Science and Engineering, Chongqing University of Technology, Chongqing, China; 3 School of Physics and Energy, Xuzhou University of Technology, Xuzhou, China; 4 Division of Nanomaterials and Jiangxi Key Lab of Carbonene Materials, Jiangxi Institute of Nanotechnology, Nanchang, China; 5 Shanghai Synchrotron Radiation Facility (SSRF), Shanghai Advanced Research Institute, Chinese Academy of Sciences, Beijing, China; 6 Department of Chemical Engineering, Waterloo Institute for Nanotechnology, Waterloo Institute for Sustainable Energy, University of Waterloo, Waterloo, ON Canada

**Keywords:** Electrocatalysis, Structural properties, Electrocatalysis, Catalytic mechanisms

## Abstract

The lack of available protons severely lowers the activity of alkaline hydrogen evolution reaction process than that in acids, which can be efficiently accelerated by tuning the coverage and chemical environment of protons on catalyst surface. However, the cycling of active sites by proton transfer is largely dependent on the utilization of noble metal catalysts because of the appealing electronic interaction between noble metal atoms and protons. Herein, an all-non-noble W/WO_2_ metallic heterostructure serving as an efficient solid-acid catalyst exhibits remarkable hydrogen evolution reaction performance with an ultra-low overpotential of −35 mV at −10 mA/cm^2^ and a small Tafel slope (−34 mV/dec), as well as long-term durability of hydrogen production (>50 h) at current densities of −10 and −50 mA/cm^2^ in alkaline electrolyte. Multiple in situ and ex situ spectroscopy characterizations combining with first-principle density functional theory calculations discover that a dynamic proton-concentrated surface can be constructed on W/WO_2_ solid-acid catalyst under ultra-low overpotentials, which enables W/WO_2_ catalyzing alkaline hydrogen production to follow a kinetically fast Volmer-Tafel pathway with two neighboring protons recombining into a hydrogen molecule. Our strategy of solid-acid catalyst and utilization of multiple spectroscopy characterizations may provide an interesting route for designing advanced all-non-noble catalytic system towards boosting hydrogen evolution reaction performance in alkaline electrolyte.

## Abstract (web summary)

The high cost and low abundance of noble metals largely restrict practical applications for electrochemical hydrogen production. Here, the authors prepare ultrasmall tungsten nanoparticles on metallic tungsten dioxide nanorods and demonstrate excellent activities for the alkaline hydrogen evolution reaction.

## Introduction

As a clean and sustainable energy carrier, hydrogen is one of the most promising alternatives to traditional fossil fuels for addressing global energy crisis and environmental pollution^1^. One of the most economical and effective strategy of hydrogen production is electrocatalytic hydrogen evolution reaction (HER), which is driven by electricity from sustainable energies (i.e., solar and wind) without any emission of carbon dioxide, satisfying the mission of global carbon neutrality^2,3^. Alkaline HER process can avoid the acidic corrosion and dissolution issues of catalysts, and achieve high-purity hydrogen gas (>99.7%), showing an attractive and extensive application^4,5^. HER electrocatalysis usually demands the usage of noble-metal-based catalysts to lower the applied overpotentials, but the high cost and low reserve have restricted their widespread utilization. Moreover, compared to the direct proton-coupled electron reaction (${{{{{{\rm{2H}}}}}}}^{\ast }+{{{{{{\rm{2e}}}}}}}^{-}\to {{{{{{\rm{H}}}}}}}_{2}+\ast$, where ∗represents the active site) in acidic electrolyte, it is worth noting that an additional water dissociation step (${{{{{{\rm{H}}}}}}}_{2}{{{{{\rm{O}}}}}}+\ast+{{{{{{\rm{e}}}}}}}^{-}\to {{{{{{\rm{H}}}}}}}^{\ast }+{{{{{{\rm{OH}}}}}}}^{-}$, also known as Volmer step) is required to produce available protons before hydrogen generation in alkaline HER process^6^, while noble platinum catalyst is kinetically inefficient for the cleavage of H-OH bonds^7^, usually leading to two or three orders of magnitude lower activity of alkaline HER process than that in acids^8^. Therefore, the exploration of cost-effective catalysts towards efficiently breaking H-OH bonds for proton generation is valuable and significant in alkaline HER process.

Transition metal oxides have long been advocated as highly efficient HER catalysts due to their flexible chemical and electronic structures with fascinating physical and chemical properties^9^. In particular, tungsten (W), remarkable for its complex electronic structure featuring open d and f shells^10^, can form rich oxidation states ranging from +6 to 0 (WO_3-x_, 0 ≤ x < 3)^11^. Tungsten oxides with substoichiometric phases have been demonstrated as potential alternatives to commercial platinum catalyst in acidic HER process^12–14^, benefiting from these abundant oxygen vacancies that can afford substoichiometric WO_3-x_ catalysts with favorable hydrogen adsorption energies and improved conductivity^15^. However, their alkaline HER activities have been rarely explored, because tungsten oxides featured with acidic-oxide property will be gradually dissolved in alkaline electrolyte^16–18^. Although first-row (3d) transition metals (Fe, Co, Ni) as additives have been proven to significantly improve the alkaline HER performance of tungsten oxides^19^, the introduction of foreign atoms probably obscures the catalytic mechanism, since the identification of doping state of low-atomic-number 3d metals among tungsten arrays is rather challenging^20^. Therefore, it is still unclear that whether tungsten-oxide-dominant materials can be employed as stable catalysts for highly-efficient and stable alkaline HER process, when the local chemical environment of W atoms is altered.

To this end, we have focused on a special form of W/WO_2_ metallic heterostructure. Unlike previously reported acid-like catalyst surface constructed by metal-oxide matrix supported noble-metals^18,21^, where metal oxides are expected to provide an acid-like catalyst surface under neutral or alkaline conditions, while noble metals are essentially required as active sites due to the energy-favorable interactions of noble-metal and produced protons on catalyst surface^22^. The W/WO_2_ metallic heterostructure can spontaneously construct a dynamic proton-concentrated surface only by tungsten-atom itself, thus enabling kinetically fast HER process in high-pH conditions, which can be understood in the viewpoint of solid-acid catalyst: (i) featured by unusual metallic and acidic-oxide properties, the WO_2_ component can serve as highly-active Lewis acid sites for the adsorption and cleavage of H_2_O molecules^23,24^ (proton generation), facilitating the formation of hydrogen tungsten bronze (H_*x*_WO_*y*_) intermediates^25^ (proton storage); (ii) the in-situ generated H_*x*_WO_*y*_ intermediates are considered as Brønsted acid sites with reversible adsorption/desorption behaviors of protons^26,27^; (iii) considering the relatively sluggish hydrogen desorption kinetics of H_x_WO_y_ intermediates, the introduction of zero-valence W (W^0^) sites can further accelerate the deprotonation kinetics of Brønsted acids for the cycling of active sites due to the optimized electronic interactions between W^0^ atoms and protons at the W/WO_2_ interface^28^ (proton donation and regeneration of active sites). In addition, compared to traditional semiconducting tungsten oxides, the metallic feature of WO_2_ component can also afford the tungsten-oxide matrix with improved alkaline leaching resistance (WO_2_ + OH^−^ → WO_4_^2−^ + H_2_O), because the aggressive hydroxyl species from produced OH^−^ intermediates and electrolyte can be rapidly repulsed from the electron-rich catalyst surface at the cathode^29,30^.

Herein, we report a feasible pyrolysis-reduction strategy to synthesize W/WO_2_ metallic heterostructure on Ni foam. Owing to the solid-acid sites with strong abilities of proton generation and reversible behaviors of hydrogen adsorption/desorption, a dynamic proton-concentrated surface is constructed on W/WO_2_ solid-acid catalyst, which enables the all-non-noble W/WO_2_ catalyst to show superior HER activity with an ultra-low overpotential of −35 mV at −10 mA/cm^2^ and a small Tafel slope (−34 mV/dec) in alkaline electrolyte. Moreover, the solid-acid catalyst is exceptionally stable in alkaline electrolyte, showing no significant activity degradation for hydrogen production at −10 and −50 mA/cm^2^ over 50 h. To the best of our knowledge, this is the first time that a tungsten-oxide-dominant material serves as a solid-acid catalyst with a remarkable HER performance in alkaline electrolyte, outperforming all tungsten/molybdenum oxides and most 3d-metal oxides reported to date.

## Results

### Morphological characterization of W/WO_2_ metallic heterostructure

W/WO_2_ metallic heterostructure was synthesized by a pyrolysis-reduction method using W_18_O_49_ nanowires (NWs) as parent materials (Supplementary Fig. 1). In brief, W_18_O_49_ NWs were homogeneously coated on the pre-treated Ni foam (Supplementary Fig. 2), followed by stirring in the mixture solution of polyethylene oxide-co-polypropylene oxide-co-polyethylene oxide (P_123_, carbon source), 2-amino-2-hydroxymethyl-propane-1,3-dio (Tris) and dopamine (DA, carbon source) for 24 h to coat organic carbon source over W_18_O_49_ materials. Afterwards, the desired W/WO_2_ metallic heterostructure was obtained by pyrolyzing the free-standing organic-tungsten precursor at 700 °C under a mixture of Ar/H_2_ (v/v = 8:1) atmosphere for 2 h (Supplementary Fig. 2), where the reduction reactions can be simplified as the equations (${{{{{\rm{C}}}}}}+{{{{{{\rm{W}}}}}}}_{18}{{{{{{\rm{O}}}}}}}_{49}\to {{{{{\rm{W}}}}}}+{{{{{{\rm{WO}}}}}}}_{2}+{{{{{{\rm{CO}}}}}}}_{2}\uparrow$, ${{{{{{\rm{H}}}}}}}_{2}+{{{{{{\rm{W}}}}}}}_{18}{{{{{{\rm{O}}}}}}}_{49}\to {{{{{\rm{W}}}}}}+{{{{{{\rm{WO}}}}}}}_{2}+{{{{{{\rm{H}}}}}}}_{2}{{{{{\rm{O}}}}}}\uparrow$). In the pyrolysis process, carbon can cause the reduction of parent high-valence W_18_O_49_ parent materials for the generation of desired W and WO_2_ products. In the following alkaline HER process, the produced graphite carbon layers not only improve the electrocatalytic charge transfer, but also alleviate the alkaline-leaching rate of inner W/WO_2_ materials. Meanwhile, the possibly formed tungsten-carbide by-products (e.g., WC, W_2_C) are excluded by X-ray photoelectron spectroscopy (XPS) and Raman characterizations (Supplementary Fig. 3). For comparison, WO_2_ nanorods and W NPs were also synthesized under different conditions, respectively (seen in the method section).

**Fig. 1:**
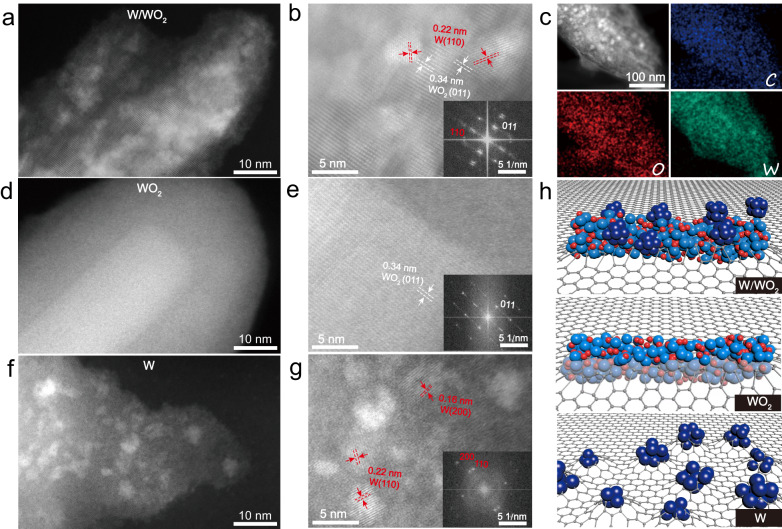
Morphological characterizations of W/WO_2_, WO_2_ and W counterparts. Low-magnification STEM images of **a** W/WO_2_, **d** WO_2_, and **f** W NPs. High-resolution STEM images of **b** W/WO_2_, **e** WO_2_, and **g** W NPs, with the corresponding FFT patterns in the insets. **c** STEM-EDS mapping of W/WO_2_ showing the homogeneous distribution of C (blue), O (red) and W (green) elements. **h** Structural illustrations of W/WO_2_ (blue and deep blue), WO_2_ (blue) and W NPs (deep blue) embedded on carbon matrix.

The crystal structure of W/WO_2_ heterostructure was verified by X-ray diffraction (XRD) pattern. As shown in Supplementary Fig. 4, besides of the predominant characteristics of underlying Ni foam, the XRD pattern of WO_2_ sample reveals a set of characteristic signals at 25.8°, 37.1°, 52.9°, and 59.7°, which are indexed to the (011), (−211), (220), and (031) facets of monoclinic structured WO_2_ phase (JCPDS No. 32–1393), respectively, while metallic W NPs present a simple cubic phase with lattice parameters of a = b = c = 3.16 Å (JCPDS no. 89–2767). W/WO_2_ metallic heterostructure shows hybrid signals containing WO_2_ and W phases, indicating the coexistence of two types of tungsten-based phases. The overall morphology of W/WO_2_ product was characterized by scanning electron microscopy (SEM). From low-magnification SEM image, the Ni skeletons are closely coated by W/WO_2_ products (Supplementary Fig. 5a, b), and the high-magnification SEM image and corresponding energy dispersive X-ray spectra (EDS) show one-dimensional (1D) nanorod structure of the as-obtained W/WO_2_ materials with radial lengths over 500 nm (Supplementary Fig. 5c, d), indicating the 1D nanostructure of tungsten-oxide precursor is still retained after high-temperature pyrolysis treatment. High-angle annular dark-field scanning transmission electron microscopy (HAADF-STEM) can allow us to observe the hybrid structure of W/WO_2_ metallic heterostructure at atomic level. As shown in Fig. 1a, high density of nanoparticles with sizes less than 10 nm are dispersed on tungsten-based nanorods, and two sets of lattice fringes with spacing values of 0.22 and 0.34 nm can be seen from the high-magnification STEM image (Fig. 1b). Combining the analysis of corresponding fast Fourier transform (FFT) pattern (inset in Fig. 1b), we can conclude that the observed lattice fringes should be attributed to the (110) and (011) facet of supported W NPs and underlying WO_2_ matrix, respectively. Meanwhile, the homogeneous distribution of C, O, and W elements over the entire W/WO_2_ metallic heterostructure is visualized by the STEM-EDS mapping images (Fig. 1c). In addition, the morphology of WO_2_ nanorods and W NPs were also examined. Figure 1d shows a typical carbon-encapsulated WO_2_ nanorod, and the corresponding high-magnification STEM image and FFT pattern evidence the single-crystalline phase of WO_2_ species (Fig. 1f). In contrast, numerous W NPs with lateral dimension below 5 nm are immobilized on carbon matrix (Supplementary Fig. 6, Fig. 1f, g), indicating the 1D W_18_O_49_ precursors have been disintegrated into ultrasmall W NPs under high-temperature reducing conditions. Therefore, based on above crystal structure and morphological characterizations, three types of tungsten-based catalysts (W, WO_2_, and W/WO_2_) are indeed obtained (Fig. 1h).

### Structural characterizations of W/WO_2_ metallic heterostructure

**Fig. 2:**
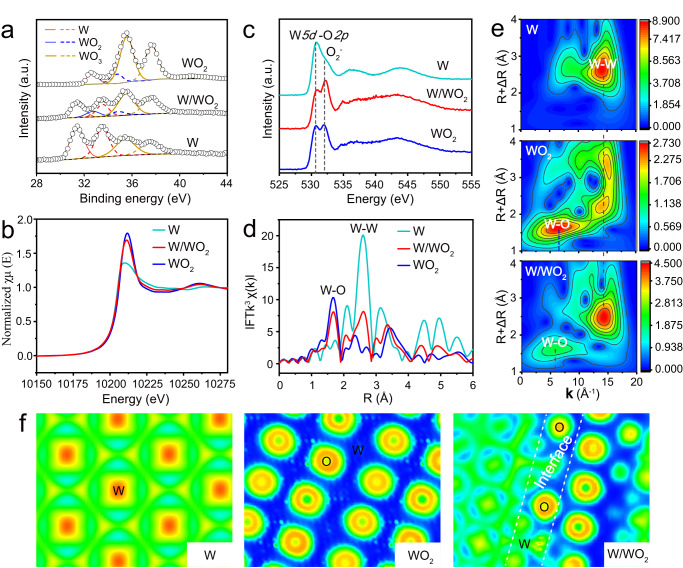
Structural characterizations of W/WO_2_, W and WO_2_ counterparts. **a** W *4* *f* core-level XPS spectra. **b** W *L*_3_-edge XANES spectra. **c** O *K*-edge NEXAFS spectra. **d** R space profiles of W (light blue), WO_2_ (blue), and W/WO_2_ (red) extracted from the corresponding W *L*_3_-edge EXAFS spectra. **e** WT-EXAFS plots. **f** The calculated ELF images of W, WO_2_, and W/WO_2_. Green to red indicates the gradually increased electron localization.

X-ray photoelectron spectroscopy (XPS) and X-ray adsorption spectroscopy (XAS) were performed to gain insight into the subtle change of the local chemical and electronic structures for the above three tungsten-based materials in. As shown in Fig. 2a, W and WO_2_ samples exhibit W^0^ and W^4+^ signals at low (31.4 eV for W^0^ *4f*_7/2_ and 33.5 eV for W^0^ *4f*_5/2_) and high (32.7 eV for W^4+^ *4f*_7/2_ and 34.8 eV for W^4+^ *4f*_5/2_) binding energies after deconvolution, respectively. In addition to the intrinsically characteristic peaks, the inevitable surface high-valence oxidation species (35.5 eV for W^6+^ *4f*_7/2_ and 37.6 eV for W^6+^ *4f*_5/2_) are also observed on W and WO_2_ samples^31^. As expected, W/WO_2_ sample contains features of both W and WO_2_ species at the same binding energies after deconvolution, thus demonstrating the coexistence of W and WO_2_ components in W/WO_2_. The normalized X-ray absorption near-edge structure (XANES) profiles of W *L*_3_-edge reveal that the white line intensity of W/WO_2_ sample is much higher than that of metallic W NPs, but slightly lower relative to that of WO_2_ counterpart (Fig. 2b), indicating the average oxidation state of tungsten atoms in W/WO_2_ is between 0 and 4 + . Also, the whole W *L*_3_-edge XANES profile of W/WO_2_ extremely resembles that of WO_2_ rather than metallic W, further suggesting that W atoms of WO_2_ and W/WO_2_ materials may have similar chemical state^32^. Correspondingly, from the viewpoint of coordinated oxygen atoms, the O K-edge near edge X-ray absorption fine structure (NEXAFS) spectroscopy can give additional information on the chemical structures of W atoms in above-mentioned tungsten species (Fig. 2c). One can see that both WO_2_ and W/WO_2_ samples exhibit split peaks located at energies ranging from 530 to 535 eV, where the peak at low energy (530.7 eV) originates from the overlapping band between W*5d* and O*2p* orbitals, corresponding to the W-O bonds, while the superoxide (O_2_^-^) species neighboring to surface oxygen vacancies should be responsible for another one at high energy (532.2 eV)^33^. Comparing to the O K-edge NEXAFS spectroscopy of WO_2_ counterpart, W/WO_2_ metallic heterostructure exhibits decreased intensity of W-O bonds but increased signal of O_2_^-^ species, indicating the formation of rich oxygen vacancies. Meanwhile, such an enrichment of oxygen vacancies in W/WO_2_ metallic heterostructure is also resolved by electron spin resonance (ESR) spectroscopy (Supplementary Fig. 7), which exhibits a strong and symmetrical ESR signal at g = 2.002, manifesting rich unpaired electrons trapped on vacancies around W centers^34^. Further, the subtle difference of local coordination structure in W, WO_2_, and W/WO_2_ samples were discriminated by the Fourier-transformed extended X-ray absorption fine structure (FT-EXAFS) spectra (R-space) (Fig. 2d). As can be seen, W sample exhibits a distinct peak at 2.6 Å, corresponding to the W-W scattering path, while a predominant peak at approximately 1.6 Å is presented on WO_2_ sample, which can be assigned to the W-O coordination. Meanwhile, we also observe a weak but still visible W-W coordination at 2.4 Å on WO_2_ sample, which is almost absent in stoichiometric WO_3_ reference (Supplementary Fig. 8), demonstrating the metallic property of WO_2_ phase. As expected, the R-space profile of W/WO_2_ heterostructure exhibits the combined features of W and WO_2_ counterparts. However, comparing to WO_2_ sample, W/WO_2_ sample exhibits a relatively weak W-O coordination but significantly increased intensity of W-W scattering path, implying the introduction of W NPs probably breaks the homogeneity of local W-O coordination structure, and further enhance the metallic property of WO_2_ matrix. Wavelet transform (WT)-EXAFS with high-resolution in both **k** and R spaces can be used to visually examine the atomic configuration of W atoms. In line with the FT-EXAFS analysis, the WT-EXAFS contour plot of W/WO_2_ sample shows an increased intensity of W-W scattering path centered at about **k** = 14.3 Å^−1^ relative to that of WO_2_ sample, further suggesting the increased metallic feature. Moreover, W/WO_2_ sample exhibits intensity maximum at **k** = 5.6 Å^−1^ for W-O coordination, which is definitely different from WO_2_ sample (**k** = 6.3 Å^−1^), suggesting the local W-O bonding configuration of WO_2_ matrix has been altered due to the introduction of W NPs. Finally, the electronic structures of three tungsten-based materials were also examined by the electron localization function (ELF) calculations. Unlike the uniform electronic structures of W and WO_2_ counterparts, W/WO_2_ model exhibits distinctly modified electronic structures at the interface, where the coexistence of electron localized (O atoms in WO_2_) and delocalized (W^0^ atoms in W metal) states can afford W/WO_2_ heterostructure with synergistic effort for kinetically fast water dissociation and hydrogen desorption steps in alkaline HER process.

### Evaluation of alkaline HER electrocatalysis using W/WO_2_ catalyst

**Fig. 3:**
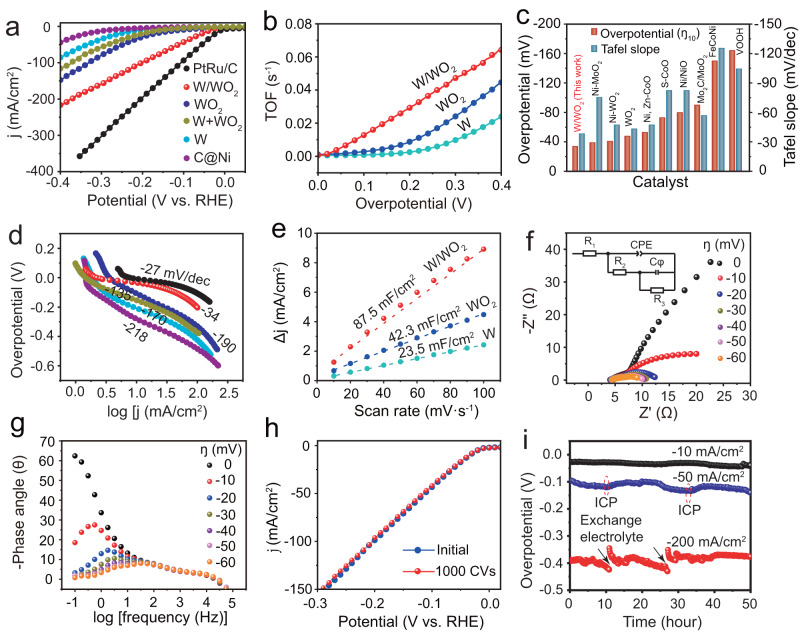
The evaluation of HER performance of W/WO_2_ solid-acid catalyst in 1.0 M KOH electrolyte (pH = 14, Rs = ~4.0 Ω, mass loading = 3.2 mg/cm^2^). **a** polarization (LSV) curves of C@Ni (wine red), W (light blue), WO_2_ (deep blue), W + WO_2_ (green), W/WO_2_ (red) and commercial PtRu@C (black) catalysts. **b** TOF plots of W, WO_2_, and W/WO_2_ catalysts at overpotentials of 0 ~ 0.4 V. **c** Comparison of overpotentials (10 mA/cm^2^) and Tafel slopes of W/WO_2_ solid-acid catalyst and previously reported excellent transition-metal-oxide based HER catalysts in alkaline electrolyte. **d** Tafel plots of C@Ni (wine red), W (light blue), WO_2_ (deep blue), W + WO_2_ (green), W/WO_2_ (red) and commercial PtRu@C (black) catalysts. **e** The determination of C_dI_ by plotting the current density variation (Δj) against the scan rates (10–100 mV s^−1^). **f** Nyquist plots (the inset shows the equivalent circuit for the simulation) and **g** the corresponding Bode phase plots of W/WO_2_ solid-acid catalyst with the increase of applied overpotentials at 0 (black), −10 (red), −20 (deep blue), −30 (green), −40 (purple), −50 (light purple), and −60 (orange) mV. **h** LSV curves of W/WO_2_ solid-acid catalyst before (deep blue) and after (red) 1000 CVs. **i** Chronopotentiometry measurements of W/WO_2_ solid-acid catalyst at current densities of −10 (black), −50 (blue), and −200 (red) mA/cm^2^, and the red dashed circle indicates the extraction of electrolyte for ICP detection.

The HER performance of free-standing W/WO_2_ solid-acid catalyst was evaluated in 1.0 M KOH electrolyte using a typical three-electrode setup, where bare carbon coated Ni foam (C@Ni), W NPs, WO_2_, and PtRu/C catalysts were also measured under the same conditions for comparison. As shown in Fig. 3a, bare C@Ni sample exhibits a negligible alkaline HER activity, although W and WO_2_ catalysts exhibit enhanced alkaline HER activities with decreased overpotentials at −10 mA/cm^2^ (W: ƞ_10_ = −183 mV; WO_2_: ƞ_10_ = −106 mV), their activities are still inferior to other W-/Mo-based catalysts and conventional 3d-metal (Fe, Co, Ni) oxides reported to date (Fig. 3c and Supplementary Table 1). Encouragingly, the as-obtained W/WO_2_ solid-acid catalyst displays a superior alkaline HER activity with value of ƞ_10_ as low as −35 mV, excelling all previously reported tungsten oxide catalysts and most state-of-the-art non-noble oxide catalysts (Fig. 3c and Supplementary Table 1), and even being comparable to commercial PtRu/C (−11 mV). In addition, the alkaline HER activity of the physical mixture of W and WO_2_ (W + WO_2_) was also examined under the same conditions, where the values of ƞ_10_ and Tafel slope are determined to be −153 mV and −135 mV/dec, respectively, and the markedly improved Tafel slope may be attributed to the synergistic effect of WO_2_ and W components for water dissociation and hydrogen desorption steps in alkaline HER process. However, the alkaline HER activity of W + WO_2_ is still inferior to W/WO_2_ catalyst, suggesting the W/WO_2_ interfaces constructed by chemical bonds can improve the alkaline HER activity intrinsically. The turnover frequency (TOF) plot of W/WO_2_ solid-acid catalyst has been calculated at overpotentials of 0 ~ 0.4 V (Fig. 3b, calculated detail seen in method section), where W/WO_2_ solid-acid catalyst exhibits a high TOF value of 0.013 s^−1^ at overpotential of −100 mV, nearly 4.6- and 15.1-fold higher than WO_2_ and W counterparts, demonstrating the high intrinsic activity of W/WO_2_ solid-acid catalyst. Further, the accelerated reaction kinetics of alkaline HER process on W/WO_2_ solid-acid catalyst was revealed by Tafel slope. W/WO_2_ solid-acid catalyst exhibits a sharply decreased Tafel slope (−34 mV/dec) relative to those of W (−170 mV/dec) and WO_2_ (−190 mV/dec) counterparts (Fig. 3d), even being comparable to the value of commercial PtRu/C catalyst (−27 mV/dec). This result indicates the high energy barrier of additional water dissociation has been substantially weakened, and the concomitant hydrogen production follows the fastest kinetics of Tafel pathway due to the coverage of rich protons on W/WO_2_ catalyst surface. Moreover, the HER performance of W, WO_2_, and W/WO_2_ powders were also examined using the rotating disk electrode technique in 1 M KOH electrolyte (Supplementary Fig. 9). As can be seen, W/WO_2_ catalyst still exhibits a remarkable alkaline HER activity with a low overpotential (−60 mV) at −10 mA/cm^2^ and a small Tafel slope (−54 mV/dec), which are more excellent than those of W and WO_2_ counterparts, and still excelling most previously reported metal oxides (Fig. 3c).

In order to better understand the proton coverage on W/WO_2_ catalyst surface, double-layer capacitance (*C*_*dI*_) and electrochemical impedance spectroscopy (EIS) of W/WO_2_ catalyst were examined, with W and WO_2_ counterparts as references. The larger value of *C*_*dI*_ usually implies the higher electrochemical surface area (ECSA) for alkaline HER process. The *C*_*dI*_ value of catalyst can be extracted from the cyclic voltammetry (CV) curves with different scan rates at non-Faradaic voltage windows (Supplementary Fig. 10). The *C*_*dI*_ value of W/WO_2_ catalyst is determined to be up to 87.5 mF/cm^2^, nearly 3.7- and 2.1-fold enhancement than those of W and WO_2_ counterparts, respectively (Fig. 3e), suggesting the construction of W/WO_2_ metallic heterostructure can provide rich active sites for the adsorption of H_2_O reactants and reaction intermediates in alkaline HER process^35^. The electrochemical impedance spectroscopy (EIS) measurements were then performed to track the accumulation of protons deriving from the activation of H_2_O reactants on W/WO_2_ catalyst surface^36^. All Nyquist plots of W/WO_2_ samples were simulated by a double-parallel equivalent circuit model in accordance with previous reports^36,37^, where R_1_ represents the uncompensated solution resistance (R_s_), the first parallel components (constant phase element (CPE) and R_2_) indicate the charge transfer resistance caused by the adsorption and activation of water molecules at low frequencies^38,39^, and the second parallel ones of R_2_ and C_φ_ are attributed to the hydrogen adsorption resistance and pseudo-capacitance at high frequencies, respectively (Fig. 3f and Supplementary Table 2). As expected, all electrochemically treated W/WO_2_ samples exhibit the similar R_1_ value (~4.0 Ω), and the small values of R_2_ for all W/WO_2_ catalysts suggest the fast charge transfer kinetics between catalyst surface and H_2_O molecules. In particular, R_2_ decreases to 4.4 Ω sharply with a negligible water diffusion resistance at applied overpotential of −20 mV, indicating the adsorption and activation of water molecules on the W/WO_2_ catalyst surface can be achieved under low overpotentials. Further, we also notice that R_3_ and C_φ_ are largely overpotential-dependent, where W/WO_2_ catalysts exhibit significantly decreased R_3_ with increased C_φ_ when increasing the applied overpotentials, in particular, the value of R_3_ can be as low as approximately 1.8 Ω, while C_φ_ is up to 0.017 F at an overpotential of −30 mV, suggesting the hydrogen adsorption resistance is very small, and the pseudo-capacitance of proton coverage is very large on W/WO_2_ catalyst surface under low overpotentials. In contrast, both W and WO_2_ counterparts show a sluggish alkaline HER kinetics with large values of R_ct_ and high negative phase angles on the Nyquist and Bode plots, respectively (Supplementary Fig. 11), suggesting a sluggish HER kinetics in an alkaline environment.

Besides of high electrocatalytic activity, catalyst durability is another significant concern for practical application. In order to evaluate the durability of the W/WO_2_ solid-acid catalyst, the as-obtained catalyst was firstly performed by 1000 cyclic voltammograms (CVs) in the voltage window from 0 to −0.2 V (versus RHE) with a scan rate of 100 mV/s. As can be seen, the HER polarization curve of W/WO_2_ almost overlaps with the original one after 1000 potential cycles (Fig. 3h), suggesting the stable proton-coupled electron redox activity of W/WO_2_ solid-acid catalyst in alkaline electrolyte. A prolonged chronopotentiometry measurement was further applied to evaluate the long-term durability of the catalysts at current densities of −10, −50 mA/cm^2^. It can be seen that the superior alkaline HER activity is well retained on W/WO_2_ solid-acid catalyst after continuous hydrogen production for more than 50 h. The relatively good stability of W/WO_2_ solid-acid catalyst in alkaline electrolyte is also confirmed by the inductively coupled plasma-optical emission spectroscopy (ICP-OES). After long-term hydrogen production, the initial electrolyte, used electrolyte, and refreshed electrolyte (indicated by red dashed circle) shows no significant increase in the concentrations of dissolved W species with values of 7.5 × 10^−6^, 9.8 × 10^−6^, and 6.3 × 10^−7 ^mol/L, respectively. In particular, the rather low concentration (6.3 × 10^−7 ^mol/L) of dissolved W species in the refreshed electrolyte directly suggests the structural robustness of W/WO_2_ catalyst after long-term hydrogen production in alkaline electrolyte. In addition, we also evaluate the stability of W/WO_2_ catalyzing alkaline HER process at a current density of −200 mA/cm^2^ (the lower limit for industrial water electrolysis). No significant activity loss can be observed on W/WO_2_ catalysts after long-term hydrogen production, suggesting the good catalytic stability at industrial current density. For the characterization of used catalyst, a rough catalyst surface with rich defects is observed on the used W/WO_2_ solid-acid catalyst (Supplementary Fig. 12), suggesting the insertion of hydrogen may alter the local chemical structure of W/WO_2_ solid-acid catalyst. Meanwhile, the enhanced signal of oxygen vacancies detected by ESR and O K-edge NEXAFS demonstrates that the proton-coupled electron reaction of HER process might have caused a slight reduction of underlying WO_2_ matrix (Supplementary Fig. 13). Therefore, based on the comprehensive evaluations of alkaline HER activity and stability on W/WO_2_ materials, the significantly improved structural robustness of our well designed W/WO_2_ composites in high-pH solutions can be attributed to the following two reasons: (i) W/WO_2_ heterostructures have intrinsically good oxidation resistance with co-existence of metal and oxide features and the protection of surface carbon layers^19,29,40,41^, meanwhile, the strong chemical and electronic interactions of W and WO_2_ components within W/WO_2_ may further improve the structural robustness in alkaline solutions^42,43^; (ii) unlike the naturally alkaline leaching under circuit potential condition, the negative potentials at cathode can provide rich electrons to avoid the oxidation and dissolution of low-valence tungsten species during alkaline HER process.

### Multiple spectroscopy characterizations discovering electrocatalytic mechanism

**Fig. 4:**
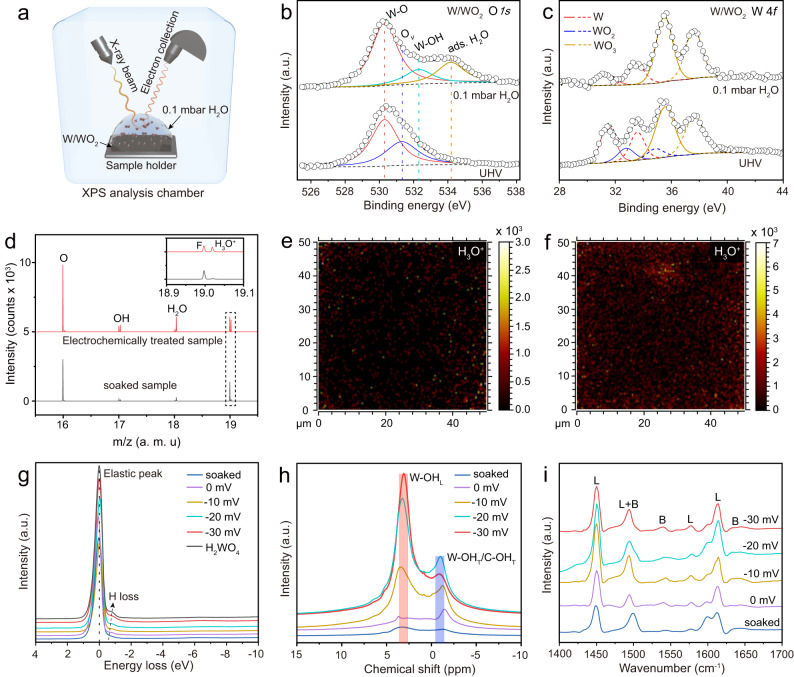
Multiple high-resolution spectroscopy characterizations tracing the coverage and chemical state of protons on W/WO_2_ solid-acid catalyst. **a** Schematic illustration of the in situ NAP-XPS measurement under 0.1 mbar H_2_O atmosphere. **b** O *1* *s* and **c** W *4* *f* XPS spectra of W/WO_2_ recorded under UHV and 0.1 mbar H_2_O conditions, respectively. **d** TOF-SIMS profiles of W/WO_2_ reference (black) and electrochemically treated sample (red), and the inset shows the enlarged signals ranging from 18.9 to 19.1, where the fluorine (F, m/z = 19.00) signal may be originated from the inevitable contamination of fluorine-containing sealing ring in the analysis chamber during Bi^3+^ cations impacting the sample, while the H_3_O^+^ species can be responsible for the higher signal at m/z = 19.02. **e** 2D distribution images of H_3_O^+^ species on W/WO_2_ only soaked in alkaline electrolyte and **f** W/WO_2_ after alkaline HER process. **g** REELS spectra, **h** ^1^H MAS NMR, and **i** Py-IR spectra of W/WO_2_ catalysts after electrochemical treatments by the gradually increased overpotentials, where soaked, 0, -10, -20, and -30 mV are represented by deep blue, pink, orange, light blue, and red colors, and the black color stands for commercial H_2_WO_4_ sample.

Comprehensive characterizations were performed to gain insight into the origin of significantly enhanced alkaline HER activity on W/WO_2_ metallic heterostructure. Firstly, the deuterated-effect-induced inferior alkaline HER activity suggests that the fast HER kinetics of water dissociation has been lowered on W/WO_2_ catalyst surface because of the larger zero-point energy of OD* relative to the OH* intermediates (difference of ~1400 cal) (Supplementary Fig. 14a)^44^. Besides, we also observe that the deuterated effect has more negative influence on the Tafel slope of W/WO_2_ catalyst relative to W and WO_2_ counterparts (Supplementary Fig. 14b), because the sluggish kinetics of water dissociation hampers the proton coverage on W/WO_2_ catalyst surface, which severely lowers subsequent hydrogen desorption kinetics. Secondly, near ambient pressure X-ray photoelectron spectroscopy (NAP-XPS) measurement was performed to investigate the adsorption and cleavage of H_2_O molecules on W, WO_2_, and W/WO_2_ catalyst surface under a water pressure of 0.1 mbar (Fig. 4a), with the core-level XPS spectroscopy recorded under an ultrahigh vacuum (UHV) condition as a reference^8^. For the referenced O *1* *s* XPS profile of W/WO_2_ sample under an UHV condition. It exhibits two peaks at 530.3 and 531.3 eV after deconvolution (Fig. 4b), corresponding to the lattice oxygen (W-O) species and oxygen vacancies (O_*v*_), respectively^45^. After introducing 0.1 mbar H_2_O molecules (Supplementary Fig. 15), W/WO_2_ metallic heterostructure exhibits a vanished O_*v*_ signal but significantly enhanced concentrations of W-OH (532.3 eV) and H_2_O (533.3 eV) species at higher binding energies (Supplementary Table 3), indicating O_*v*_ have served as the adsorption sites of H_2_O molecules, and the cleavage of H-OH bonds is easily proceeded on W/WO_2_ catalyst surface. Also, such an accelerated water dissociation kinetics of W/WO_2_ catalyst has been confirmed by the obvious decrease of low-valence tungsten species in the corresponding W *4* *f* core-level XPS spectra (Fig. 4c). In contrast, W and WO_2_ counterparts display inferior activities of water dissociation kinetics (Supplementary Fig. 16 and Supplementary Table 3), in particular, only an extremely weak W-OH signal is observed on W catalyst surface after exposure in 0.1 mbar H_2_O atmosphere, because W^0^ (W) sites with more filled *d* orbitals have weaker electrostatic affinity to electron-rich H_2_O molecules than W^4+^ (WO_2_) atoms^46^.

Finally, with the clear elucidation of proton production on W/WO_2_ catalyst surface in water dissociation step, the coverage and chemical environment of produced protons determine the activity of subsequent hydrogen generation on W/WO_2_ solid-acid catalyst surface^36^. Time-of-flight secondary ion mass spectroscopy (TOF-SIMS) is very sensitive to the examine the hydrogen species after alkaline HER process. In order to eliminate the interference of adsorbed H_2_O molecules and OH^-^ anions (electrolyte), W/WO_2_ sample only soaked in KOH electrolyte was selected as a reference. We can observe that the electrochemically treated W/WO_2_ catalyst exhibits markedly increased signals of hydrogen-containing intermediates, i. e., OH, H_2_O, H_3_O^+^, in particular, the sharply increased H_3_O^+^ signal directly suggests the formation of proton-concentrated catalyst surface^21^ (Fig. 4d). In order to visually display the distribution of H_3_O^+^ on the electrochemically treated W/WO_2_ catalyst surface, two-dimensional (2D) image analysis of the soaked and electrochemically treated W/WO_2_ samples are also provided in Fig. 4e, f. Obviously, one could notice that the concentration of H_3_O^+^ species on the electrochemically treated W/WO_2_ catalyst is much higher than that of the only soaked counterpart, indicating the achievement of proton-concentrated catalyst surface on W/WO_2_ solid-acid catalyst. Multiple spectroscopy characterizations were further performed to analyze the property of the produced protons on the used W/WO_2_ catalyst surface treated by increased applied overpotentials (ƞ=0, −10, −20, −30 mV), with W/WO_2_ sample only soaked in KOH electrolyte and commercial tungstic acid (H_2_WO_4_) as the references. The reflection electron energy loss spectroscopy (REELS) can break the limitation of conventional XPS technique for the detection of H element. As shown in Fig. 4g, the REELS plot of W/WO_2_ soaked sample only exhibits a predominantly elastic peak, while an obvious H signal neighboring to elastic peak is observed on commercial H_2_WO_4_ sample^47–49^, indicating the hydrogen concentration of W/WO_2_ soaked sample is lower than the detecting limitation (molar ratio: ~20%) of REELS technique. As expected, all electrochemically treated W/WO_2_ catalysts exhibited gradually enhanced H signals when increasing the applied overpotentials (0 to −30 mV), indicating a proton-concentrated surface could be constructed on W/WO_2_ metallic heterostructure under an ultra-low overpotential, which is in good consistency with the analysis of EIS measurement. Note that the major contributor of detected H signal should be attributed to W/WO_2_ species rather than underlying carbon matrix, as evidenced by the REELS measurements of bare carbon materials after treatments under increased overpotentials (Supplementary Fig. 17). Solid-state ^1^H magic-angle-spinning nuclear magnetic resonance (^1^H MAS NMR) spectra exhibits two broad bands at approximately −1.3 and 3.4 ppm on soaked W/WO_2_ sample (Fig. 4h), in which the peak at lower chemical shift can be attributed to the mixture of terminated hydroxyl (W-OH_T_ and/or C-OH_T_) species on WO_2_ and carbon matrix, while the higher one originates from the lattice hydroxyl (W-OH_L_) species in WO_2_ matrix. Similar to REELS characterization, all electrochemically treated W/WO_2_ samples exhibit significant increased signals of W-OH_T_ and W-OH_L_ species under increasing overpotentials, indicating the increased coverage of protons over W/WO_2_ catalyst surface. Meanwhile, we also notice that the proportion of W-OH_T_/W-OH_L_ decreases with the increase of applied potentials, which may be attributed to the continuously increased coverage of produced protons. The increasing negative potentials can result in more water molecules being adsorbed on W/WO_2_ catalyst surface, and the water molecules rapidly react with electrons for the cleavage of H-OH bonds, then the produced H* intermediates insert into the lattice of tungsten oxides (W-OH_L_) for constructing proton-concentrated catalyst surface with electronic and chemical environments approaching to those of H_2_WO_4_ materials. While the terminated electron-rich hydroxyl species, such as the produced OH* intermediates and chemically/physically adsorbed OH^-^ molecules (W-OH_T_) cannot follow the increasing tendency because of the enhanced Coulomb effect at cathode. In addition, ^1^H MAS NMR profiles collected at overpotentials of −10, −20, and −30 mV become extremely similar to that of commercial H_2_WO_4_ reference (Supplementary Fig. 18), demonstrating the chemical environment of protons on W/WO_2_ catalyst surface is approaching to commercial H_2_WO_4_ reference. All Py-IR spectroscopy of electrochemically treated samples exhibit typical adsorption peaks in the wavenumber range of 1400 ~ 1650 cm^−1^, where peaks at 1450, 1577, and 1613 cm^−1^ are ascribed to the chemisorption of pyridine at surface Lewis acid sites (L), while peaks at 1540 and 1638 cm^−1^ indicate the presence of Brønsted acid sites (B), and the signal at 1494 cm^−1^ contains contribution of Lewis acid and Brønsted acid sites (Fig. 4i and Supplementary Fig. 19). Impressively, the electrochemically treated W/WO_2_ catalysts also exhibited enhanced concentrations of Brønsted acid sites with the increase of applied overpotentials (Supplementary Table 4), indicating the produced protons of water dissociation mainly serve as Brønsted acids with reversible behaviors of hydrogen adsorption and desorption on W/WO_2_ solid-acid catalyst surface^26,27^.

### DFT calculations

**Fig. 5:**
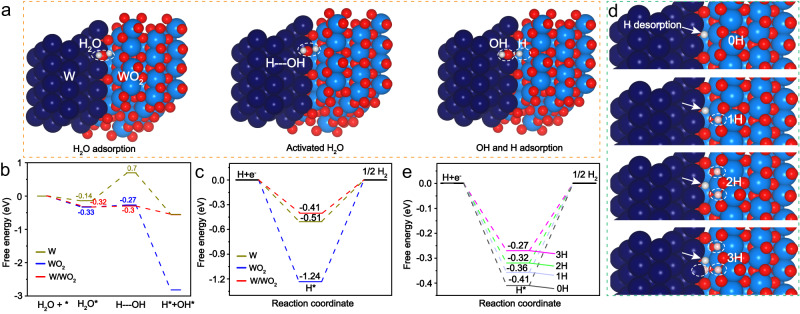
DFT calculations of alkaline HER activities on W, WO_2_, and W/WO_2_ models. **a** Schematic pathway for alkaline HER process on W/WO_2_ interface, where H_2_O molecule undergoes water adsorption, activated H_2_O adsorption, produced OH and H adsorption in alkaline HER process. The corresponding calculated free energy diagrams for **b** water adsorption and dissociation, and **c** hydrogen desorption steps on W/WO_2_ interface, W (110) and WO_2_ (01-1) facets, respectively. **d** H desorption (white) models and **e** the corresponding calculated free energies tuned by continuously increased neighboring Brønsted acid sites (pink). Panel **a** and **d** are created by VESTA software^54^.

Such a significant enhancement of alkaline HER activity of W/WO_2_ solid-acid catalyst was also elucidated by first-principle density functional theory (DFT) calculations, with simulations of W and WO_2_ models as references (Fig. 5a, Supplementary Fig. 20). Specifically, more detailed front, side and top structures of W/WO_2_ interface were also calculated in Supplementary Fig. 21. WO_2_ (−0.33 eV) and W/WO_2_ (−0.32 eV) materials exhibit more negative adsorption energies of H_2_O molecules than that of W sample (−0.14 eV) (Fig. 5b), which suggests the WO_2_ and W/WO_2_ catalyst surfaces are beneficial for the adsorption and activation of H_2_O reactants^50,51^. As expected, W exhibits a sluggish kinetics of prior water dissociation step with energy barrier (ΔG_H2O_) up to 0.84 eV, while the activation energy barrier of H_2_O molecules can be sharply reduced to 0.06 eV on WO_2_ model (Fig. 5b), indicating oxygen-vacancy-rich WO_2_ is generally effective for the cleavage of H-OH bonds. Encouragingly, the energy barrier of water dissociation is further decreased to 0.02 eV on W/WO_2_ catalyst surface (Supplementary Table 5), excelling most previously reported noble metal alloy catalysts^52,53^. With an ultra-low energy barrier of water dissociation benefiting the formation of available protons on W/WO_2_ catalyst surface, the following proton detachment will determine the reaction rate of alkaline HER process, where hydrogen adsorption free energy (ΔG_H_) of H* intermediates is a good descriptor to evaluate the HER activity, and the optimum ΔG_H_ is usually around thermoneutral value (0)^22^. In contrast to the calculations of water dissociation, W catalyst exhibits a lower hydrogen adsorption energy (−0.51 eV) than that of WO_2_ catalyst (−1.24 eV) (Fig. 5c), implying zero-valence W sites are more beneficial for hydrogen desorption. Accordingly, the calculated ΔG_H_ (−0.41 eV) decreases sharply when coupling W and WO_2_ components as heterostructures (Fig. 5c), indicative of significantly improved hydrogen desorption kinetics on W/WO_2_ catalyst surface. Encouragingly, we observe that the ΔG_H_ value (−0.27 eV) of W/WO_2_ is extremely closer to thermoneutral value (0) after the W/WO_2_ interface inserted by H^+^ atoms (W/H_*x*_WO_*y*_) (Fig. 5d, e), indicating the neighboring Brønsted acid site can further improve the hydrogen desorption kinetics on W/WO_2_ catalyst surface. Moreover, the hydrogen desorption activities of W/WO_2_ interface can be further tuned by the amounts of neighboring Brønsted acid site (Fig. 5e), demonstrating the coverage of Brønsted acids also plays a vital role in the hydrogen desorption step, which agrees well with aforementioned experimental characterizations (Figs. 3a, e, f and 4d, e, f).

**Fig. 6:**
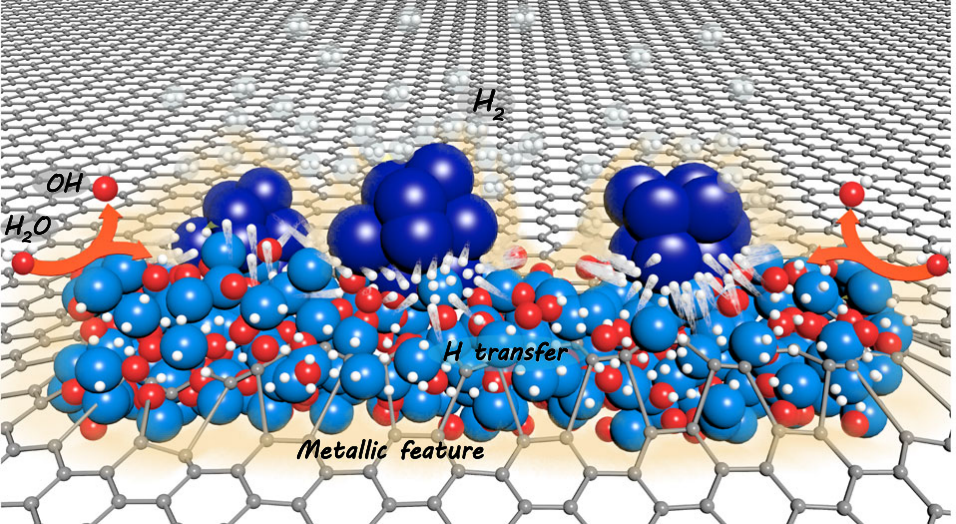
Schematic illustration of the dynamic proton-concentrated catalyst surface. The cleavage of water reactants is proceeded on WO_2_ surface for the generation of protons (light blue surface), where the aggressive OH^-^ species can be rapidly repulsed from the electron-rich catalyst surface (orange layer), while the highly active protons prefer to transfer to the interface of WO_2_ (light blue) and W (deep blue) components for the proton recombination and release of hydrogen gas.

Based on above comprehensive characterizations in solid experimental evidences and theoretical simulations, a well-defined dynamic proton-concentrated surface is constructed on W/WO_2_ solid-acid catalyst, where the dynamic feature of proton-concentrated surface facilitates the proton transfer for the cycling of active sites on catalyst surface (Fig. 6). First, the oxygen-vacancy-rich WO_2_ component mainly serves as highly active Lewis acid sites for the adsorption and cleavage of H_2_O molecules (proton generation). Second, protons can be enriched on W/WO_2_ catalyst surface under ultra-low overpotentials due to the strong hydrogen storage ability of H*x*WO*y* intermediates (Brønsted acid sites, proton storage). Finally, the appealing electronic interaction between zero-valence W atoms and protons accelerates the deprotonation kinetics of Brønsted acids for the cycling of active sites (proton donation and regeneration of active sites).

## Discussion

In summary, a facial pyrolysis-reduction method is used to prepare free-standing W/WO_2_ solid-acid catalyst on Ni foam. Owing to the in situ constructed solid-acid catalyst surface with reversible adsorption/desorption behaviors of protons in alkaline HER process. The well-designed W/WO_2_ heterostructure catalyzing hydrogen production follows the kinetically fast Volmer-Tafel pathway with an ultra-low overptential at −10 mA/cm^2^ and a small Tafel slope (−34 mV/dec), as well as a long-term electrocatalytic stability (>50 h) in alkaline electrolyte, outperforming all tungsten/molybdenum oxide catalysts and most of *3d*-metal oxides reported to date. Moreover, multiple spectroscopy characterizations combined with DFT calculations discover that the dynamic proton-concentrated surface can be constructed on W/WO_2_ metallic heterostructure under low overpotentials, enabling W/WO_2_ catalyzing alkaline hydrogen production to follow kinetically fast Volmer-Tafel pathway. Our strategy of solid-acid catalyst and utilization of multiple spectroscopy characterizations may provide a valuable route for designing advanced all-non-noble catalytic system towards boosting HER performance in alkaline electrolyte.

## Methods

### Chemicals

Tungsten chloride (WCl_6_ 99%, Aladdin, USA), Polyvinylpyrrolidone (PVP, Macklin, China), polyethylene oxide-*co*-polypropylene oxide-*co*-polyethylene oxide (P_123_ 98%, Adamas Reagent, China), dopamine hydrochloride (DA 98%, Aladdin, USA), and 2-amino-2-hydroxymethyl-propane-1,3-diol (Tris 98%, Aladdin, USA).

### Preparation of W_18_O_49_ NWs

In a typical procedure, a mixture of WCl_6_ (500 mg) and PVP (20 mg) precursors was dissolved in absolute ethanol (50 mL), and a homogeneous yellow solution was obtained after stirring for 30 min. Afterwards, the obtained solutions were transferred into a 100 mL Teflon-lined autoclave and heated at 180 °C for 24 h. The resultant blue W_18_O_49_ product was collected after purification and centrifugation.

### Preparation of W_18_O_49_@Ni foam

The Ni foam (2 cm × 4 cm) was treated by 0.1 M HCl solutions, and washed with absolute ethanol and distilled water before drying at 60 °C. A total of 50 mg W_18_O_49_ powder was re-dispersed into 2 mL ethanol by sonication, and the homogeneous ink was dropped onto the pre-treated Ni foam (2 cm × 4 cm). In order to better lock the W_18_O_49_ precursor, Ni foam supported W_18_O_49_ powder was pressed into a thinner foil structure (Supplementary Fig. 3).

### Preparation of W_18_O_49_@PDA@Ni foam

The fabricated W_18_O_49_@Ni foam was immersed into mixture solutions (250 mL) of DA (100 mg), P_123_ (160 mg), and Tris (40 mg), and W_18_O_49_@PDA@Ni foam was obtained after stirring for 24 h. In addition, W_18_O_49_ powder was also added into the same mixture solutions containing DA, P_123_, and Tris components with continuous stirring for 24 h. After that, W_18_O_49_@PDA powder was collected by purification and centrifugation.

### Preparation of W/WO_2_ metallic heterostructure

Both W/WO_2_ powder and free-standing electrode were prepared in accordance with the following thermal treatment. The desired W/WO_2_ metallic heterostructure was obtained by pyrolyzing the W_18_O_49_@PDA@Ni foam (W_18_O_49_@PDA powder) precursor at 700 °C under a mixture of Ar/H_2_ (v/v = 8:1) atmosphere for 2 h. For comparison, power and free-standing types of WO_2_ sample were synthesized at 700 °C under a pure Ar atmosphere for 2 h, while W sample was collected at high temperature of 750 °C under a mixture of Ar/H_2_ (v/v = 8:1) atmosphere for 2 h. The total loading mass of supported materials (W/WO_2_ and C, 32 mg) can be determined by the mass difference of bare Ni foam (2 × 4 cm, 250 mg) and Ni foam supported W/WO_2_ materials (2 × 4 cm, 282 mg). The loading mass of tungsten element was 3.2 mg/cm^2^ in accordance with the ICP-MS analysis (W: 79.1 wt%).

### Characterization

XRD pattern was recorded on a Bruker AXS D8 Advance X-ray diffractometer with a Cu Ka radiation target (40 V, 40 A). TEM characterization was performed using an FEI Tecnai G2F20 microscope. Atomic-level HAADF-STEM images and the corresponding STEM-EDS elemental maps were measured on an FEI Titan Themis Z 3.1 equipped with a SCOR spherical aberration corrector and a monochromator. The probe convergence angle was 80 mrad, and camera length was 115 mm in the STEM mode. The loading mass of W atoms in W/WO_2_ materials and the dissolved W species were measured by the ICP-OES measurements (Agilent ICP-OES730). O K-edge NEXAFS spectra was performed at the Catalysis and Surface Science End-station at the BL11U beamline in the National Synchrotron Radiation Laboratory (NSRL) in Hefei, China. W L_3_-edge X-ray adsorption spectra including XANES and EXAFS profiles were collected at BL11B station at the Shanghai Synchrotron Radiation Facility (SSRF).

For NAP-XPS characterization, in situ NAP-XPS measurements were conducted on a SPECS NAP-XPS instrument, where the photon source is the monochromatic X-ray source of Al Kα (1486.6 eV), and the overall spectra resolution is Ag 3d5/2, <0.5 eV FWHM at 20 kcps@UHV. The dried sample was transferred to the analysis chamber of XPS, and then W *4* *f*, C *1* *s*, O *1* *s* XPS profiles were simultaneously recorded under the pressure of 1 × 10^−9^ mbar. After introducing water molecules, the corresponding XPS signals were collected under the water atmosphere with a pressure of 1 × 10^−1^ mbar.

For TOF-SIMS characterization, TOF-SIMS measurement was performed on a TOF-SIMS 5–100 instrument (ION-TOF GmbH) using a 30-keV Bi^3+^ as analysis beam for negative and positive polarity measurements. The analysis beam current, raster size, and incident angles of all beam are 0.7 pA, 50 × 50 μm^2^, and 45°, respectively.

For REELS characterization, the electrochemically treated materials were dried, and then transferred to the analysis chamber for REELS characterization using ESCALAB Xi equipment (Thermo Scientific). In detail, approximately 1000 eV electrons were incident on the surface of samples, the XPS analyzer would investigate the elastic and non-elastic scattering, corresponded to the elastic signal and energy loss peak (H loss). In our measurements, all parameters, such as the total acquisition time, energy step size, number of energy steps, and number of scans were set to be 2 mins, 0.02 eV, 1001, and 4, respectively.

For NMR characterization, ^1^H Magic angle spinning nuclear magnetic resonance (MAS NMR) spectra was collected on a Bruker AVANCE NEO 400 MHz Wide Bore spectrometer operating at a magnetic field of 9.40 T. The chemical shifts for ^1^H MAS NMR spectra was referenced to tetramethy (TMS). ^1^H MAS NMR spectra was acquired at a spinning rate of 15 kHz with a π/2 pulse width of 3.5 μs and s recycle of 5 s.

For Py-IR characterization, Py-IR measurements were performed on a Nicolet 380 FT-IR instrument (Thermo Co., USA). The catalyst sample was pretreated at 353 K for 2 h under a vacuum condition, and background spectra were recorded in the ranging of 1700–1400 cm^−1^ before adsorbing pyridine molecules. After reaching the adsorption equilibrium, the catalyst sample was retreated at 353 K for 4 h under a vacuum condition for the complete remove of physically adsorbed molecules, and the Py-IR spectra of pyridine chemisorption was obtained.

### Evaluation of HER performance

Electrochemical measurements were conducted on a CHI760E electrochemical station (Shanghai Chenhua Co., China) using a standard three-electrode system in 1 M KOH electrolyte, where free-standing W/WO_2_ electrode (0.5 cm × 1.0 cm, area immersed in electrolyte is 0.5 cm × 0.5 cm), Ag/AgCl electrode, and a carbon rod were used as working, reference, and counter electrodes, respectively. For the fabrication of free-standing electrode for the physical mixture of W and WO_2_ powders (tungsten loading mass: 3.2 mg/cm^2^), W (5 mg) and WO_2_ (5 mg) were dispersed into in a mixture of 800 µL ethanol, 170 µL water, and 30 µL nafion, followed by sonication treatment at least 30 min for preparation of the homogeneous catalyst ink. Then, 200 µL of the mixture catalyst ink was dropped onto the pre-treated Ni and pressed into a thinner foil for the following electrocatalytic measurement. For the HER measurement of commercial PtRu/C catalyst, 3.2 mg of PtRu/C powder was dispersed in a mixture of of 800 μL ethanol, 170 μL water, and 30 μL Nafion, followed by sonication for at least 30 min to form a homogeneous catalyst ink. Then, all the catalyst ink was dropped onto the pre-treated Ni foam and pressed into a thinner foil for further measurement. For the preparation of W, WO_2_, W/WO_2_ catalyst ink, 5 mg of the powder materials is dispersed in the mixture of 800 µL ethanol, 170 µL water, and 30 µL nafion, followed by sonication treatment at least 30 min for the preparation of homogeneous catalyst ink. Then, 20 µL of the catalyst ink was dropped on the polished glassy carbon rotating disk electrode for electrocatalytic measurements at 1600 rpm. All potentials were recorded against the Ag/AgCl electrode and calibrated with respect to a reversible hydrogen electrode (RHE) in accordance with the Eq. (1):

1 $${{{{{{\rm{E}}}}}}}_{{{{{{\rm{RHE}}}}}}}={{{{{{\rm{E}}}}}}}_{{{{{{\rm{Ag}}}}}}/{{{{{\rm{AgCl}}}}}}}+1.02{{{{{\rm{V}}}}}}$$

### TOF calculations

The H_2_ conversion efficiencies of W, WO_2_, and W/WO_2_ can be evaluated from the TOF values, which were obtained according to the following Eq. (2):

2 $${{{{{\rm{TOF}}}}}}({{{{{\rm{s}}}}}}^{-1})=\frac{{{{{{\rm{j}}}}}}}{{{{{{\rm{2Fn}}}}}}}$$

where *j* (A) is the current at a given overpotential, 2 is the number of electrons consumed to form 1 mol H_2_, *F* represents the Faraday constant (96500 C/mol), n (mol) is the number of moles of loaded metals, which can be evaluated based on the analysis of ICP-OES measurements (W wt%: W (80.8%), WO_2_ (78.7%), W/WO_2_ (79.1%))。

### DFT calculations

All computations were performed by applying the plane-wave-based DFT method as implemented in the Vienna Ab Initio Simulation Package (VASP) and periodic slab models. The projector augmented wave potentials with the Perdew–Burke–Ernzerhof form of the exchange-correlation functional were employed in all the simulations with the energy cut-off of 450 eV, and the long-range van der Waals (vdW) interaction was described with the DFT-D3 method. The *k*-point meshes were generated using the VASPKIT tool with the grid separation of 0.04 Å^−1^ for the geometry optimizations and self-consistent field energy calculations, in which the total energy convergence and interaction force were set to be 10^−6 ^eV and 10^−2 ^eV/Å, respectively.

Based on the experimental TEM analysis, the structures of W and WO_2_ substrates were built by cleaving the (110) plane of bulk W and (01-1) bulk WO_2_, respectively. A vacuum region of 15 Å was set along the z direction to avoid the interaction between periodic images. Since the lattice parameters of WO_2_ (01-1) plane were 7.38 Å and 5.58 Å, a 2 × 2 supercell was built as the WO_2_ substrate for constructing the W/WO_2_ interface. The (110) plane of W with the lattice parameter of 2.70 Å was enlarged to build a 3 × 4 supercell, which was then cleaved (100) surface to build a W slab whose (110) and (110) surfaces were exposed. The W/WO_2_ interface was constructed by placing the W slab on the WO_2_ substrate. The lattice mismatch between W slab (10.8 Å) and WO_2_ substrate (11.2 Å) was only about 3%. The structures of constructed W/WO_2_ interface with front, side, and top images can be observed in Supplementary Fig. 21.

For evaluating the H_2_O dissociation energy barrier, the transitional state was located using the Nudged Elastic Band method. The free energy of adsorbed H (ΔG_H_) on surfaces is expressed as Eq. (3):

3 $${\Delta {{{{{\rm{G}}}}}}}_{{{{{{\rm{H}}}}}}}={\Delta {{{{{\rm{E}}}}}}}_{{{{{{\rm{H}}}}}}}+{\Delta {{{{{\rm{E}}}}}}}_{{{{{{\rm{ZPE}}}}}}}-{{{{{\rm{T}}}}}}\Delta {{{{{\rm{S}}}}}}$$

where ΔE_H_ is the hydrogen adsorption energy, ΔE_ZPE_ and ΔS are the zero point energy difference and the entropy difference between the adsorbed state and the gas phase, respectively, and T is the system temperature (298.15 K).

### Supplementary information

Supplementary Information

Peer Review File

### Source data

Source Data

## Data Availability

Data reported herein have been deposited in the Figshare database, and are accessible through 10.6084/m9.figshare.23578770. Source data are provided with this paper.

## References

[1] Zhang J (2017). Efficient hydrogen production on MoNi_4_ electrocatalysts with fast water dissociation kinetics. Nat. Commun..

[2] Yu ZY (2021). Clean and affordable hydrogen fuel from alkaline water splitting: past, recent progress, and future prospects. Adv. Mater..

[3] Luo YT (2022). Recent advances in design of electrocatalysts for high-current-density water splitting. Adv. Mater..

[4] Wang J (2017). Non-noble metal-based carbon composites in hydrogen evolution reaction: fundamentals to applications. Adv. Mater..

[5] Zhang L (2019). Cable-like Ru/WNO@C nanowires for simultaneous high-efficiency hydrogen evolution and low-energy consumption chlor-alkali electrolysis. Energy Environ. Sci..

[6] Zhu J (2020). Recent advances in electrocatalytic hydrogen evolution using nanoparticles. Chem. Rev..

[7] Subbaraman R (2011). Enhancing hydrogen evolution activity in water splitting by tailoring Li^+^-Ni(OH)_2_-Pt interfaces. Science.

[8] Dinh CT (2019). Multi-site electrocatalysts for hydrogen evolution in neutral media by destabilization of water molecules. Nat. Energy.

[9] Zhu YL (2020). Metal oxide-based materials as an emerging family of hydrogen evolution electrocatalysts. Energy Environ. Sci..

[10] Goya P (2011). W for tungsten and wolfram. Nat. Chem..

[11] Chen ZG (2019). Coordination-controlled single-atom tungsten as a non-3d-metal oxygen reduction reaction electrocatalyst with ultrahigh mass activity. Nano Energy.

[12] Wu R (2015). Metallic WO_2_-carbon mesoporous nanowires as highly efficient electrocatalysts for hydrogen evolution reaction. J. Am. Chem. Soc..

[13] Li YH (2015). Local atomic structure modulations activate metal oxide as electrocatalyst for hydrogen evolution in acidic water. Nat. Commun..

[14] Jing SY (2018). Carbon-encapsulated WO_x_ hybrids as efficient catalysts for hydrogen evolution. Adv. Mater..

[15] Zheng TT (2017). Conductive tungsten oxide nanosheets for highly efficient hydrogen evolution. Nano Lett..

[16] Pan JB (2020). A high-performance electrochromic device assembled with hexagonal WO_3_ and NiO/PB composite nanosheet electrodes towards energy storage smart window. Sol. Energy Mater. Sol. Cells.

[17] Can F (2021). Tungsten-based catalysts for environmental applications. Catalysts.

[18] Chen JD (2022). Reversible hydrogen spillover in Ru-WO_3-x_ enhances hydrogen evolution activity in neutral pH water splitting. Nat. Commun..

[19] Liu JC (2022). Optimizing hydrogen adsorption by d-d orbital modulation for efficient hydrogen evolution catalysis. Adv. Energy Mater..

[20] Li S (2021). Oxygen-evolving catalytic atoms on metal carbides. Nat. Mater..

[21] Tan H (2022). Engineering a local acid-like environment in alkaline medium for efficient hydrogen evolution reaction. Nat. Commun..

[22] Greeley J (2006). Computational high-throughput screening of electrocatalytic materials for hydrogen evolution. Nat. Mater..

[23] Jiang XL (2021). The heterostructure of Ru_2_P/WO_3_/NPC synergistically promotes H_2_O dissociation for improved hydrogen evolution. Angew. Chem. Int. Ed..

[24] Rasmussen MJ (2020). Role of tungsten modifiers in bimetallic catalysts for enhanced hydrodeoxygenation activity and selectivity. Catal. Sci. Technol..

[25] Park JY (2019). Investigation of the support effect in atomically dispersed Pt on WO_3-x_ for utilization of Pt in the hydrogen evolution reaction. Angew. Chem. Int. Ed..

[26] Fu JY (2022). Modulating the dynamics of Brønsted acid sites on PtWO_x_ inverse catalyst. Nat. Catal..

[27] Mehdad A (2018). Effect of steam and CO_2_ on ethane activation over Zn-ZSM-5. Catal. Sci. Technol..

[28] Wang FH (2022). Robust porous WC-based self-supported ceramic electrodes for high current density hydrogen evolution reaction. Adv. Sci..

[29] He CY (2018). A highly sensitive and stable SERS substrate using hybrid tungsten dioxide/carbon ultrathin nanowire beams. J. Mater. Chem. C.

[30] Chen ZG (2022). Thermal migration towards constructing W-W dual-sites for boosted alkaline hydrogen evolution reaction. Nat. Commun..

[31] Lu XF (2019). Ultrafine dual-phased carbide nanocrystals confined in porous nitrogen-doped carbon dodecahedrons for efficient hydrogen evolution reaction. Adv. Mater..

[32] Wei YS (2020). Fabricating dual-atom Iron catalysts for efficient oxygen evolution reaction: a heteroatom modulator approach. Angew. Chem. Int. Ed..

[33] Wang XY (2019). Atomic-scale insights into surface lattice oxygen activation at the spinel/perovskite interface of Co_3_O_4_/La_0.3_Sr_0.7_CoO_3_. Angew. Chem. Int. Ed..

[34] Zhang N (2016). Oxide defect engineering enables to couple solar energy into oxygen activation. J. Am. Chem. Soc..

[35] Chen ZG (2020). Eutectoid-structured WC/W_2_C heterostructures: a new platform for long-term alkaline hydrogen evolution reaction at low overpotentials. Nano Energy.

[36] Li JY (2019). Ethylene-glycol ligand environment facilitates highly efficient hydrogen evolution of Pt/CoP through proton concentration and hydrogen spillover. Energy Environ. Sci..

[37] Damian A (2006). Ni and Ni Mo hydrogen evolution electrocatalysts electrodeposited in a polyaniline matrix. J. Power Sources.

[38] Chen W (2021). Deciphering the alternating synergy between interlayer Pt single-atom and NiFe layered double hydroxide for overall water splitting. Energy Environ. Sci..

[39] Alexandria RC (2020). Electrochemical impedance spectroscopy of metal oxide electrodes for energy applications. ACS Appl. Energy Mater..

[40] Li ZC (2022). Native Ligand carbonization renders common platinum nanoparticles highly durable for electrocatalytic oxygen reduction: annealing temperature matters. Adv. Mater..

[41] Ma Z (2020). NbO_x_ nano-nail with a Pt head embedded in carbon as a highly active and durable oxygen reduction catalyst. Nano Energy.

[42] Yuan LP (2020). Molecularly engineered strong metal oxide-support interaction enables highly efficient and stable CO_2_ electroreduction. ACS Catal..

[43] Zeng X (2019). Mesoporous TiO_2_ nanospheres loaded with highly dispersed Pd nanoparticles for pH-universal hydrogen evolution reaction. Mater. Today Nano.

[44] Minamimoto H (2019). In-situ observation of isotopic hydrogen evolution reactions using electrochemical mass spectroscopy to evaluate surface morphological effect. Electrochim. Acta.

[45] Qian KC (2019). Directional oxygen activation by oxygen-vacancy-rich WO_2_ nanorods for superb hydrogen evolution via formaldehyde reforming. J. Mater. Chem. A.

[46] Yan JQ (2019). Single atom tungsten doped ultrathin α-Ni(OH)_2_ for enhanced electrocatalytic water oxidation. Nat. Commun..

[47] Rico VJ (2006). Determination of the hydrogen content in diamond-like carbon and polymeric thin films by reflection electron energy loss spectroscopy. Diam. Relat. Mater..

[48] Yubero F (2009). Identification of hydrogen and deuterium at the surface of water ice by reflection electron energy loss spectroscopy. Appl. Phys. Lett..

[49] Ren B (2016). Thermal stability of hydrogenated diamond films in nitrogen ambience studied by reflection electron energy spectroscopy and X-ray photoelectron spectroscopy. Appl. Surf. Sci..

[50] Zhang ZC (2017). Evoking ordered vacancies in metallic nanostructures toward a vacated barlow packing for high-performance hydrogen evolution. Sci. Adv..

[51] Wang LQ (2022). Rapid complete reconfiguration induced actual active species for industrial hydrogen evolution reaction. Nat. Commun..

[52] Li GW (2019). Dirac nodal arc semimetal PtSn_4_: An ideal platform for understanding surface properties and catalysis. Angew. Chem. Int. Ed..

[53] Chen LW (2021). Structurally ordered intermetallic Ir_3_V electrocatalysts for alkaline hydrogen evolution reaction. Nano Energy.

[54] Momma K (2011). VESTA 3 for three-dimensional visualization of crystal, volumetric and morphology data. J.  Appl.  Crystallogr..

