# Supplementary material for: Metallic W/WO2 solid-acid catalyst boosts hydrogen evolution reaction in alkaline electrolyte
Source: Nat Commun. 2023 Sep 2;14:5363. doi: 10.1038/s41467-023-41097-w (PMC10475068; doi:10.1038/s41467-023-41097-w)
Supplement: Supplementary file 1 — Supplementary Information [file 41467_2023_41097_MOESM1_ESM.pdf]

Supplementary Information

Metallic W/WO_2 solid-acid catalyst boosts hydrogen evolution reaction in alkaline electrolyte

Zhigang Chen[1,2†], Wenbin Gong[3,4†], Juan Wang[5†], Shuang Hou[1], Guang Yang[1], Chengfeng Zhu[1], Xiyue Fan[1], Yifan Li[1], Rui Gao[6], Yi Cui[1*]

[1]i-lab, Vacuum Interconnected Nanotech Workstation (Nano-X), Suzhou Institute of Nano-Tech and Nano-Bionics, Chinese Academy of Sciences, Suzhou 215123, China.

[2]School of Materials Science and Engineering, Chongqing University of Technology, Chongqing 400054, China.

[3]School of Physics and Energy, Xuzhou, University of Technology, Xuzhou 221018, China.

[4]Division of Nanomaterials and Jiangxi Key Lab of Carbonene Materials, Jiangxi Institute of Nanotechnology, Nanchang 330200, China.

[5]Shanghai Synchrotron Radiation Facility (SSRF), Shanghai Advanced Research Institute, Chinese Academy of Sciences, 201204, China.

[6]Department of Chemical Engineering, Waterloo Institute for Nanotechnology, Waterloo Institute for Sustainable Energy, University of Waterloo, Waterloo, Ontario N2L 3G1, Canada.

[†]These authors contributed equally to this work.

*Corresponding authors: Yi Cui, email: ycui2015@sinano.ac.cn

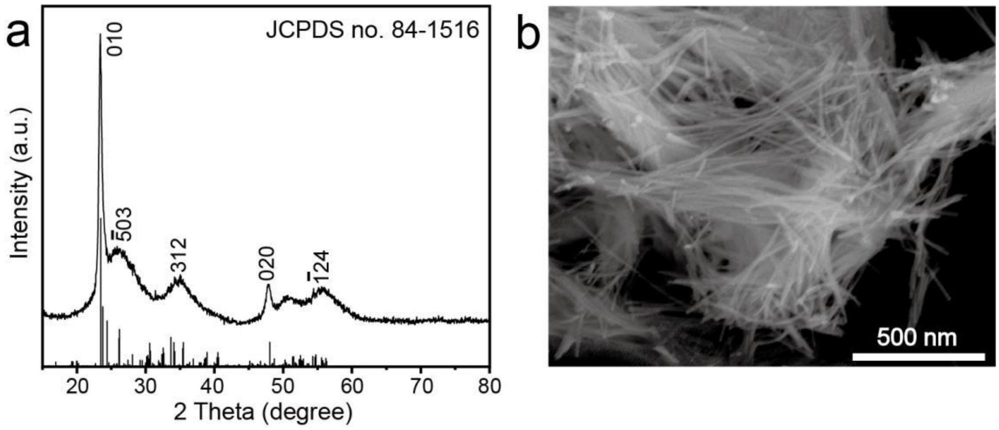

Supplementary Figure 1. Structural and morphological characterizations of $W_{18}O_{49}$ nanowires. (a) XRD pattern and (b) Typical scanning electron microscopy (SEM) image of $W_{18}O_{49}$ nanowires.

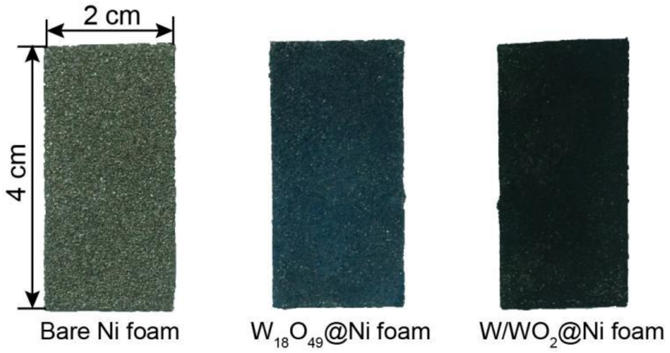

Supplementary Figure 2. Optical images of the as-prepared electrodes. Optical observations of the electrodes during the different stage of preparation procedures, with the dimensions (2 cm x 4 cm) labeled, where the homogeneous colors suggests the uniformity of supported active materials on Ni foam.

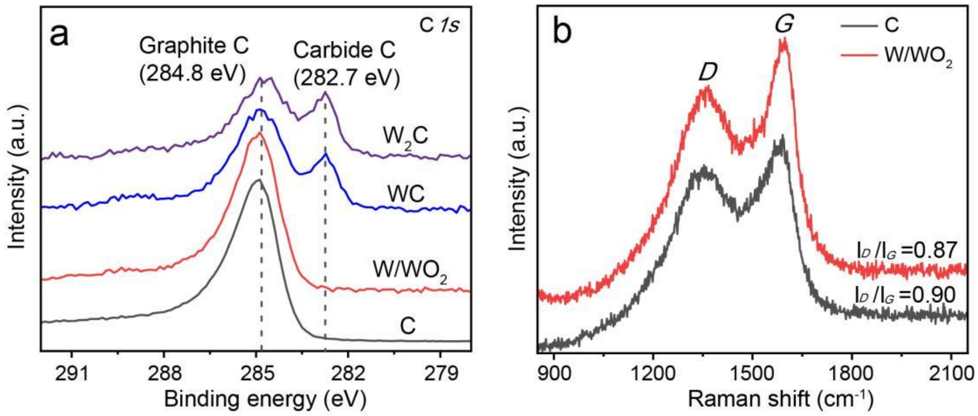

Supplementary Figure 3. The identification of surface carbon species in the as-prepared W/WO_2 materials. (a) C *1s* XPS and (b) Raman spectra.

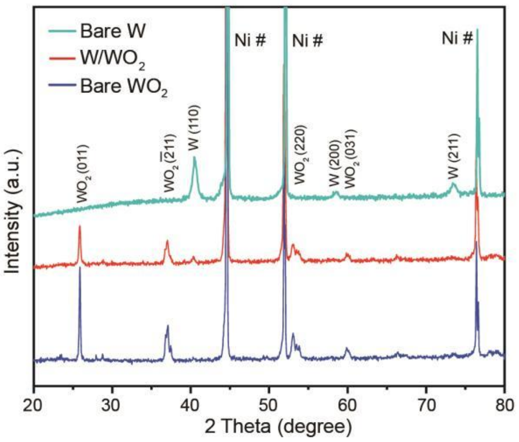

Supplementary Figure 4. Structural characterizations of different tungsten-based materials. XRD patterns of W, WO_2, and W/WO_2.

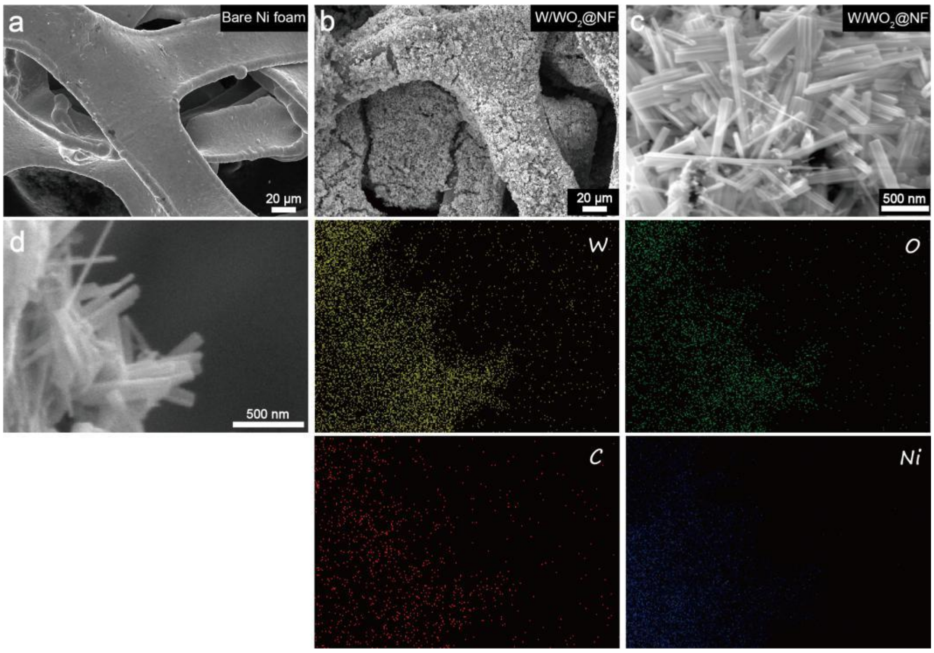

Supplementary Figure 5. Morphological characterizations of W/WO$_2$@Ni foam. (a) SEM image of bare Ni foam. (b) Low- and (c) high-magnification SEM images of W/WO$_2$ materials on Ni foam. (d) Typical nano-rod structure of W/WO$_2$ materials and corresponding EDS-Mapping images of W, C, O, and Ni elements.

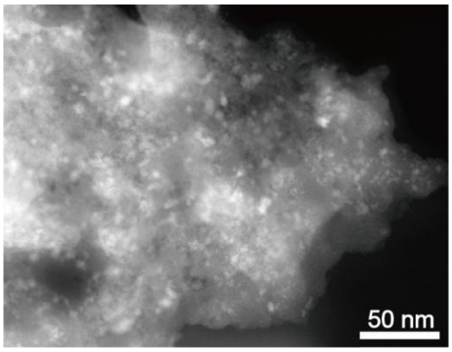

Supplementary Figure 6. Morphological characterization of W nanoparticles embedded on carbon matrix. Low-magnification scanning transmission electron microscopy (STEM) image of W nanoparticles.

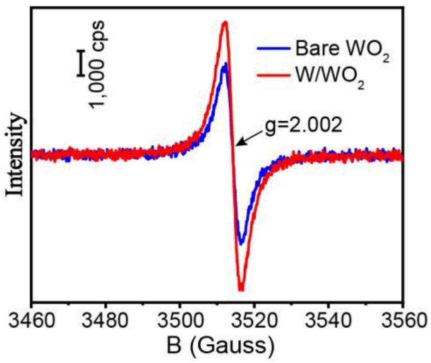

Supplementary Figure 7. Detection of rich oxygen vacancies in W/WO_2 materials. Electron spin resonance (ESR) spectroscopy of phase-pure WO_2 and W/WO_2 metallic heterostructure.

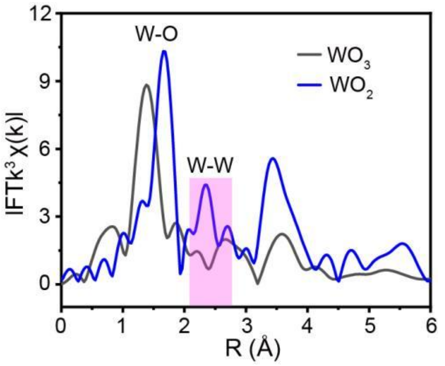

Supplementary Figure 8. Comparison of the metallic feature between WO_3 and WO_2 materials. Fourier-transformed extended X-ray absorption fine structure (FT-EXAFS) spectra (R-space) of WO_3 and WO_2 samples, where the W-W coordination at approximately 2.4 Å (pink area) can be observed on WO_2 sample, but it is almost absent on stoichiometry WO_3 reference, demonstrating the metallic property of WO_2 phase.

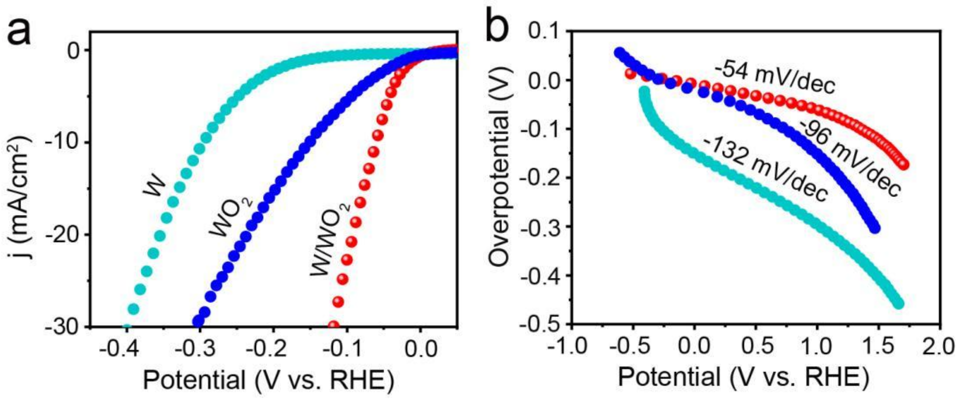

Supplementary Figure 9. The evaluation of HER performance of W, WO_2, and W/WO_2 catalysts in 1 M KOH electrolyte using the rotating disk electrode technique at 1600 rpm. (a) Polarization curve. (b) Tafel plots.

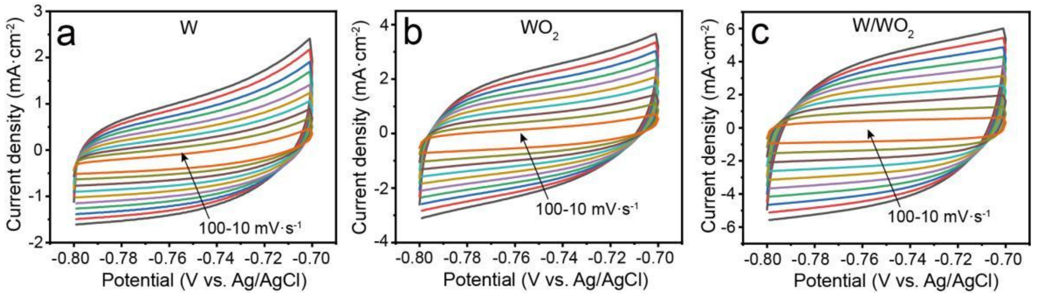

Supplementary Figure 10. Cyclic voltammetry (CV) measurements at different scan rates. (a) W, (b) WO_2, and (c) W-SAs in the voltage window from -0.8 to -0.7 V vs. Ag/AgCl at different scan rates (10-100 mV/s) in 1.0 M KOH electrolyte.

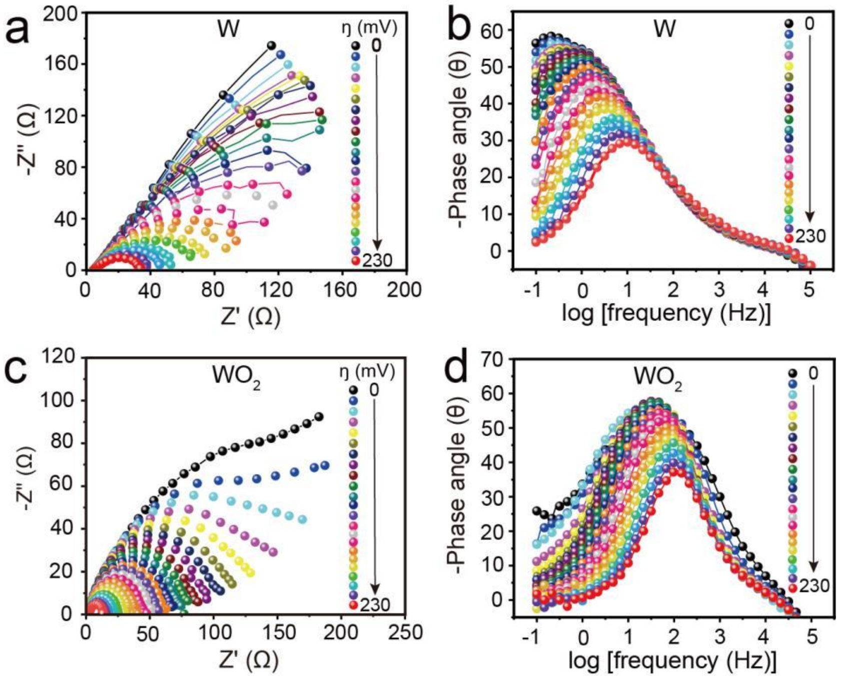

Supplementary Figure 11. Electrochemical impedance spectra (EIS) measurements of W and WO_2 catalysts. Nyquist plots of (a) W and (c) WO_2. Bode phase plots of (b) W and (d) WO_2.

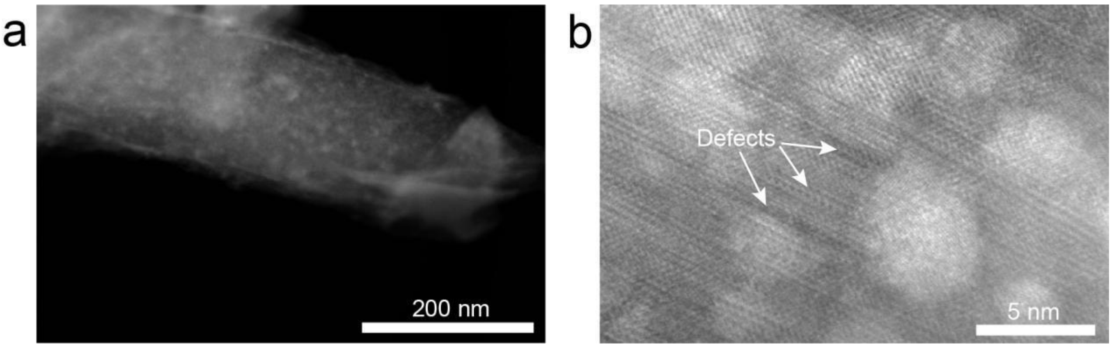

Supplementary Figure 12. Morphological characterization of used W/WO_2 catalyst. (a) Low- and (b) high-magnification STEM images of used W/WO_2 catalyst. From the low-magnification STEM image, one can see that high density of nanoparticles are still retained on the used W/WO_2 surface, while the high-magnification STEM image reveal the appearance of defects, indicating the proton-coupled electron reaction of HER process has caused a slight reduction of WO_2 matrix.

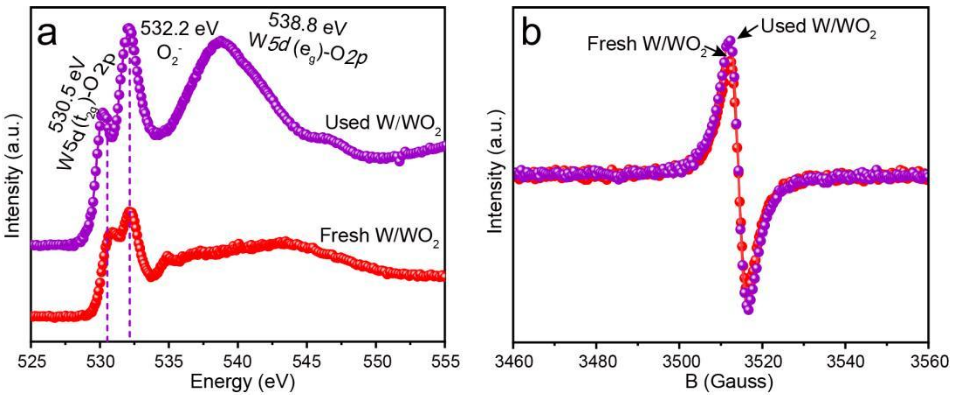

Supplementary Figure 13. Oxygen vacancy characterization of fresh and used W/WO_2 catalysts. (a) O *K*-edge near edge X-ray absorption fine structure (NEXAFS) spectroscopy. (b) ESR spectroscopy.

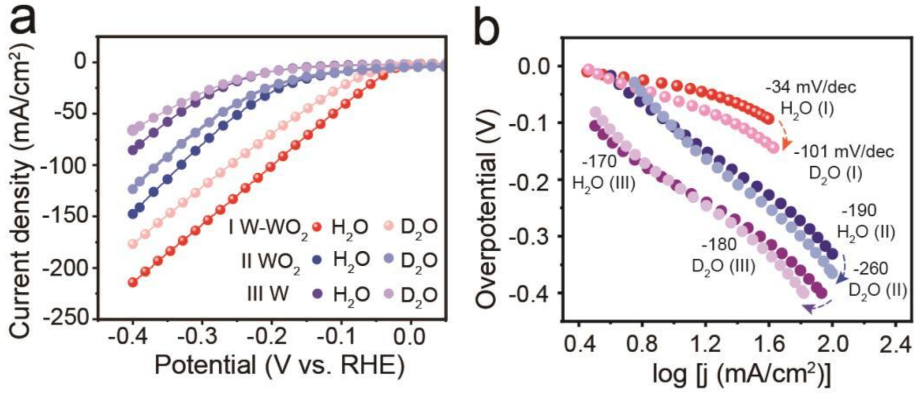

Supplementary Figure 14. Evaluation of HER activities for W, WO_2, and W/WO_2 catalysts under 1.0 M KOH/H_2O and KOD/D_2O solutions, respectively. (a) LSV curves and (b) Tafel plots of W, WO_2, W/WO_2 catalysts.

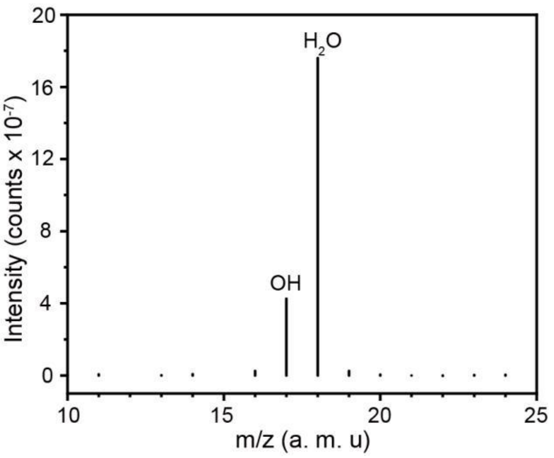

Supplementary Figure 15. Environment identification of XPS analysis chamber. H_2O mass spectra of XPS analysis chamber after introducing 0.1 mbar H_2O.

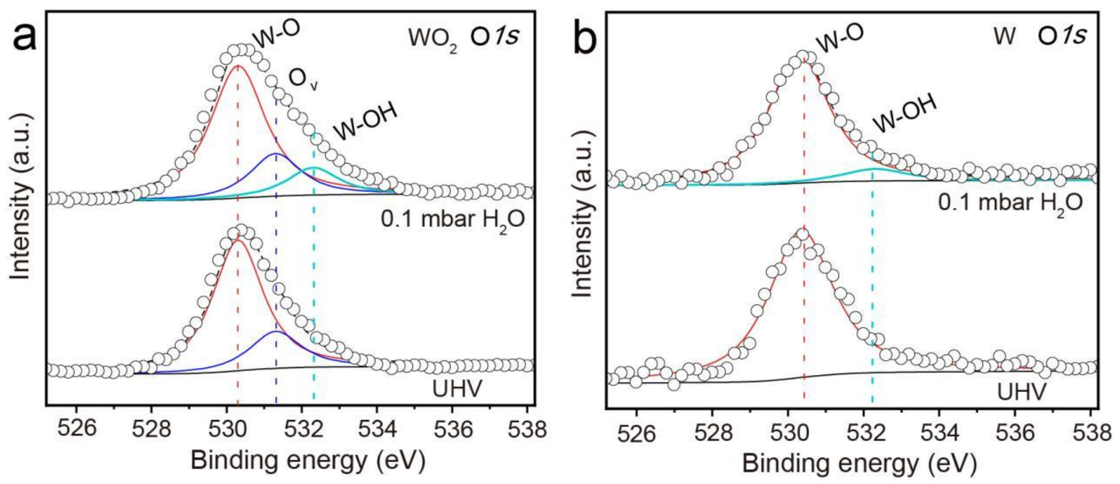

Supplementary Figure 16. In situ NAP-XPS measurements. O *1s* core-level XPS spectra of (a) WO_2 and (b) W recorded under ultrahigh vacuum and 0.1 mbar H_2O conditions.

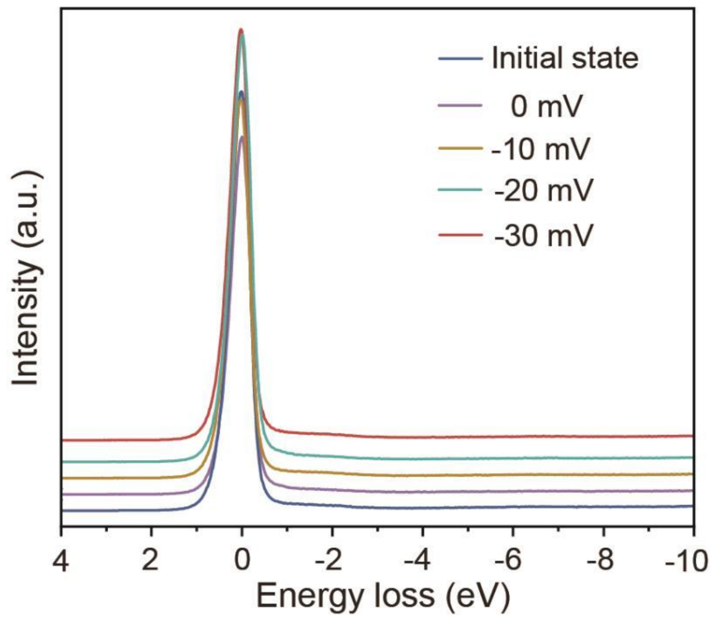

Supplementary Figure 17. Detection of H signal of bare C after treatments by increased overpotentials. The reflection electron energy loss spectroscopy (REELS) of bare C after treatments by the applied increased overpotentials, with bare C sample only soaked in KOH solutions as the initial state.

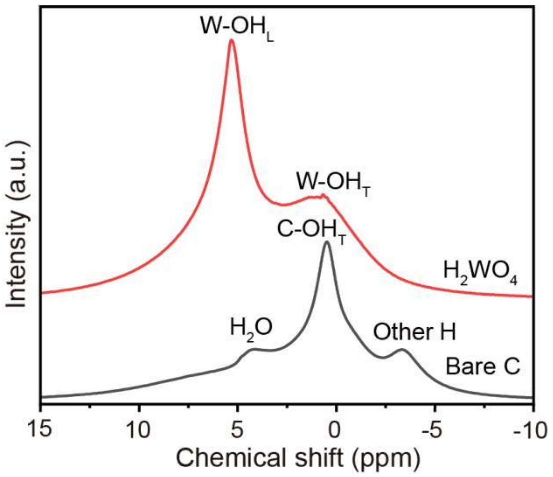

Supplementary Figure 18. Identification of chemical states of hydrogen species in bare C and commercial H_2WO_4 references. Solid-state ^1H magic-angle-spinning nuclear magnetic resonance (^1H MAS NMR) spectra of bare C and commercial H_2WO_4 materials. For commercial H_2WO_4 sample, two continuous hydrogen peaks can be observed at chemical shifts of 5.3 and 0.8 ppm, where hydrogen signal at 5.3 ppm can be attributed to lattice hydrogen (W-OH$_L$), while another one at 0.8 ppm may originate from the terminated hydrogen species (W-OH$_T$). For bare C sample, one predominant peak with two shoulder bands are visible at 4.3, 0.4, and -3.3 ppm, where the predominant peak (0.4 ppm) can be assigned to terminated hydrogen species (C-OH$_L$), while adsorbed H_2O molecules and other hydrogen species should be responsible for peaks at 4.3 and -3.3 ppm, respectively.

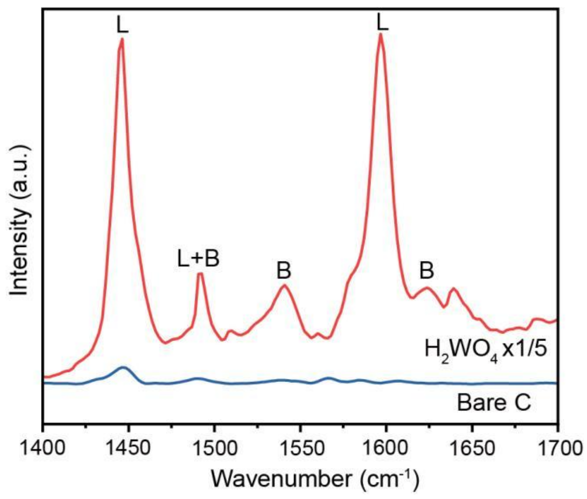

Supplementary Figure 19. Identification of chemical states of hydrogen species in bare C and commercial H_2WO_4 references. Pyridine infrared (Py-IR) spectra of bare C and commercial H_2WO_4 materials. In contrast to commercial H_2WO_4 reference, bare C exhibits weak feature of solid-acid catalyst with negligible signals of Lewis and Brønsted acid sites in the wavenumber range of 1400~1700 cm^{-1}.

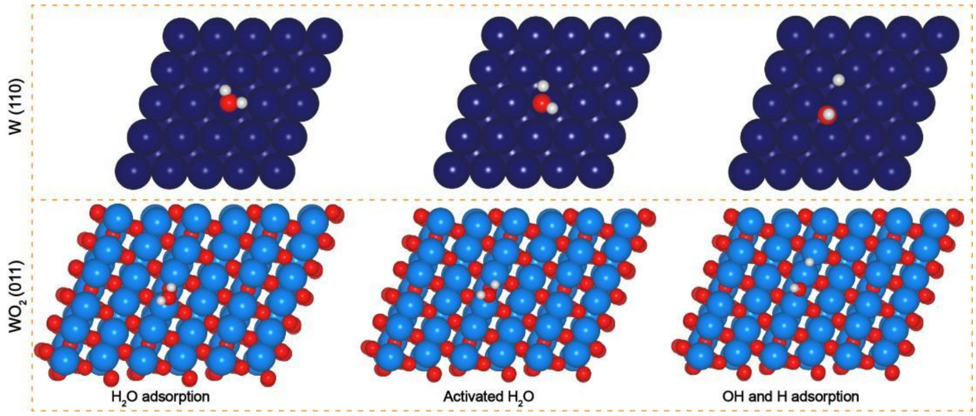

Supplementary Figure 20. Schematic pathway of alkaline HER process on W (110) and WO_2 (01-1) facets. H_2O molecule undergoes water adsorption, activated H_2O adsorption, produced OH and H adsorption in alkaline HER process. The figure is created using VESTA software (Ref: *J. Appl. Crystallogr.*, **44**, 1272-1276 (2011)).

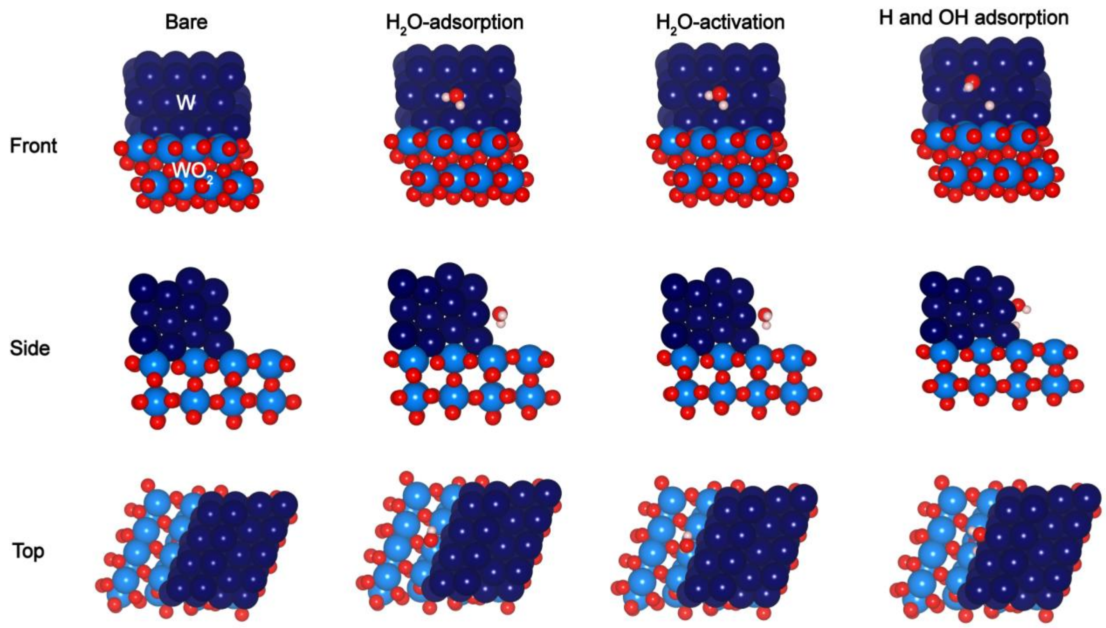

Supplementary Figure 21. Front, side and top images of the calculated structure for W/WO_2 interface. H_2O molecule undergoes water adsorption, activated H_2O adsorption, produced H and OH adsorption on W/WO_2 interface in alkaline HER process. The figure is created using VESTA software (Ref: *J. Appl. Crystallogr.*, **44**, 1272-1276 (2011)).

Supplementary Table 1. Comparison of overpotentials (10 mA/cm^2) and Tafel slopes of W/WO_2 catalyst and previously reported excellent transition-metal-oxide based HER catalysts in alkaline solutions.

Materials	Morphology	Overpotential (η_{10}, mA/cm^2)	Tafel slope (mV·dec^{-1})	Reference
mMoO_3	Nanoparticle (50-100 nm)	-138	-56	*Adv. Energy Mater*. **2016**, 6, 1600528
O*v*-α-MoO_3	Nanoparticle (~40 nm)	-138	-60	*J. Mater. Chem. A*, **2019**, 7, 257-268
GDY/MoO_3	Nanoparticle (~3.45 nm)	-170	-70	*J. Am. Chem. Soc*. **2021**, 143, 8720-8730
Co-$WO_{2.7-x}$	Nanorod	-59	-86	*Chem. Eng. J.* **2023**, 451, 138939
$La_{0.5}Sr_{0.5}CoO_{3-\delta}$	Nanoparticle	~230	-34	*Nat. Commun*. **2019**, 10, 1723
Ni/NiO-3.8	Nanoparticle (~3.8 nm)	-90	-41	*Natl Sci Rev*, **2020**, 7, 27-36
Ni-MoO_2/NF	Nanoparticle (200-300 nm)	-49	-75.1	*Appl. Catal. B Environ*. **2022**, 301, 120818
$Co_2Mo_3O_8$	Nanoparticle (5-10 nm)	-37	-58	*Nano Energy* **2021**, 87, 106217
MoS_2/Ni_2O_3H	Heterostructure	-84	-82.3	*Small* **2020**, 16, 2002212
NiO/Ni	Nanoparticle (~ 3 nm)	-121	-88	*Nano Energy* **2017**, 35, 207-214
CoO_x@CN	Nanoparticle (~ 3 nm)	-232	-115	*J. Am. Chem. Soc*. **2015**, 137, 2688-2694
$Ni(OH)_2$/MoS_2	Heterostructure	-80	-60	*Nano Energy* **2017**, 37, 74-80
Co@NCNT/$CoMo_yO_x$	Heterostructure	-94	-76	*J. Mater. Chem. A*, **2022**, 10, 3953-3962
CuCo/$CuCoO_x$	Heterostructure	-115	-55	*Adv. Funct. Mater*. **2018**, 28, 1704447

Supplementary Table 2. The fitted parameters of EIS data from W/WO_2 catalyst treated by increasing applied overpotentials. Note that inhomogeneities in the surface of metal oxide electrodes usually result in non-ideal capacitance in the double-layer at the solid/electrolyte interface. Thus, CPEs (CPE-T and CPE-P) are routinely used in place of pure capacitors to model this interfacial layer.

Catalyst	η (mV)	R_1 (Ω)	R_2 (Ω)	R_3 (Ω)	$C_φ$ (F)	CPE-T (F)	CPE-P (F)
	0	4.2	7.8	786.4	0.014	0.020	0.58
	-10	4.0	7.8	30.7	0.014	0.037	0.44
	-20	4.0	4.4	4.8	0.015	0.018	0.50
W/WO_2	-30	4.2	4.4	2.6	0.016	0.015	0.51
	-40	4.3	4.4	1.8	0.017	0.017	0.49
	-50	4.2	4.4	1.6	0.018	0.017	0.46
	-60	4.3	4.4	1.3	0.020	0.020	0.43

Supplementary Table 3. The relative concentrations of W-O, oxygen vacancies (O*v*), W-OH, and adsorbed H_2O in W/WO_2, WO_2, W materials under ultrahigh vacuum and 0.1 mbar H_2O conditions in accordance with the deconvolution of O *1s* XPS spectra.

Materials	Environment	W-O (at%)	O*v* (at%)	W-OH (at%)	H_2O (at%)
W/WO_2	UHV	69.5	30.5	0	0
	0.1 mbar H_2O	61.5	0	18.4	20.1
WO_2	UHV	74.9	25.1	0	0
	0.1 mbar H_2O	67.7	15.8	16.5	0
W	UHV	100	0	0	0
	0.1 mbar H_2O	91.2	0	8.8	0

Supplementary Table 4. The concentrations of Brønsted acid sites (C_B, mmol·g^{-1}) for used W/WO_2 samples treated by increased overpotentials, with the soaked sample as a reference.

State (mV)	soaked	0	10	20	30
C_B	3.6	9.9	12.3	14.5	18.2

Supplementary Table 5. The bond lengths between oxygen and hydrogen atom within H_2O molecule before and after dissociation on W, WO_2, W/WO_2 catalyst surface.

State	Bond length between O and dissociated H (Å)		
	W	WO_2	W/WO_2
Before	0.98	1.02	1.02
After	2.98	2.84	2.75

Supplementary Discussion

Supplementary Discussion 1: the identification of carbon species in the as-prepared W/WO_2 materials (Supplementary Fig. 3).

X-ray photoelectron spectroscopy (XPS) is very sensitive to identify the surface carbon species in carbon supported active materials (Ref: *ACS Nano* **9**, 5125-5134 (2015)). In addition to the predominant graphite carbon peak at high binding energy of 284.8 eV, commercial WC and W_2C powders show a sharp carbide C *1s* signal at a lower binding energy (~282.7 eV) (Supplementary Fig. 3a), but it is absolutely absent in W/WO_2 and pure carbon materials, suggesting the absence of tungsten carbide species in the as-prepared W/WO_2 materials. Raman spectra was used to evaluate the quality of graphite carbon in accordance with the D/G ratio of W/WO_2 materials. The D/G ratio is determined to be 0.87, which is even lower than that of the as-prepared bare carbon materials (D/G=0.9) (Supplementary Fig. 3b), indicating the as-prepared W/WO_2 materials are supported by relatively high-quality graphite-carbon substrates" have been added in the revised manuscript.

Supplementary Discussion 2: the attribution of a new NEXAFS peak at approximately 540 eV on the used W/WO_2 solid-acid catalyst (Supplementary Fig. 13).

In addition to the change of peak at low photo energy (~532.3 eV), we also observe that the used W/WO_2 sample exhibits a markedly enhanced band at approximately 538.8 eV, such a distinct peak can be attributed to the electronic interactions between O *2p* and metal sp orbitals in traditional 3d metal (Mn, Fe, Co, Ni) oxides (Ref: *Angew. Chem. Int. Ed.* **58**, 11720-11725 (2019)), whereas the hybridization (O *2p*-W *5d* (e_g)) between O *2p* and W *5d* (e_g) orbitals should be responsible for the appeared signal at approximately 538.8 eV in non-3d metal oxides (Ref: *Ionics* **4**, 101-105 (1998)). Moreover, the sharply increased intensity of O *2p*-W *5d* (e_g) hybridization directly suggests partially covalent interaction between oxygen and hydrogen atoms, confirming the insertion of produced hydrogen atoms into tungsten-oxide lattices after alkaline HER process.

Supplementary Discussion 3: by comparing Figure 4b and Figure S16, it can be observed that both pure-phase W and WO_2 do not have the signals of ads. H_2O.

Compared to the distinct ads. H_2O signal of W/WO_2 sample, no relevant signals can be observed on W and WO_2 sample, which can be understood by two reasons: (i) the high-density interfaces of W/WO_2 with rich unsaturated sites are expected to serve as the ideal adsorbed sites for H_2O molecules (Ref: *Nat. Commun*. **9**, 1809 (2018)), which implies that W/WO_2 heterostructure catalyst can provide more available active sites for water adsorption in comparison with W and WO_2 counterparts; (ii) it should be noted that the detection sensitivity of NAP-XPS is almost one order of magnitude lower than that of traditional ultrahigh vacuum XPS (UHV-XPS), the relatively lower detection ability may cause the negligence of weak ads. H_2O signal of W and WO_2 materials, meanwhile, the water atmosphere may further inhibits the collection of photoelectron signals from W and WO_2 catalyst surface during in situ NAP-XPS measurements.

Supplementary Discussion 4: the small energy barrier of water dissociation on W/WO_2 interface cannot be originated from the shorter bond length between oxygen and dissociated hydrogen atom within H_2O molecule.

The bond lengths between oxygen and dissociated hydrogen atom (O···H) on W, WO_2, and W/WO_2 catalyst surface are 2.98, 2.84, and 2.75 Å, respectively (Supplementary Table 5). Generally, the differences in O···H bond lengths on above three-types tungsten-based catalyst surfaces are very small, and the O···H bond length of W/WO_2 interface appears much shorter than the other two cases mainly due to the drawing perspective. Therefore, the major contribution of low activation barrier of H_2O molecules is the regulated chemical and electronic structures rather than the shorter dissociated length of O···H on W/WO_2 interface.
